# Supplementary material for: Lipidomics reveals new lipid-based lung adenocarcinoma early diagnosis model
Source: EMBO Mol Med. 2024 Mar 11;16(4):854–69. doi: 10.1038/s44321-024-00052-y (PMC11018865; doi:10.1038/s44321-024-00052-y)
Supplement: Supplementary file 1 — Appendix [file 44321_2024_52_MOESM1_ESM.pdf]

Appendix

Appendix Figure S1. Malignant epithelial cells identified in ScRNA-seq(page 2)

Appendix Figure S2. Permutation test plot for the OPLS-DA model(page 3)

Appendix Figure S3. The workflow of identifying signature lipids from untargeted lipidomics data of plasma(page 4)

Appendix Figure S4. KEGG pathways associated with PE(18:0/18:1) in TCGA-LUAD(page 5)

Appendix Figure S5. Enrichment map network of statistically significant GO categories with different concentrations PE(18:0/18:1)(page 6)

Appendix Figure S6. The distribution of 4 lipid markers(page 7)

Appendix Figure S7. The robustness of the 4 lipid markers to age(page 8)

Appendix Figure S8. Analysis of clinical information and 4 lipid markers between LUADs and HC in discovery cohort and plasma validation cohort(page 9)

Appendix Figure S9. ROC curves of lipid markers in the discovery cohort(page 10)

Appendix Table S1: Clinical information of patients used for single-cell RNA data(page 11)

Appendix Table S2.GO terms down-regulated in tumor maliganat epithelial vs nonmalignant epithelial cells in ScRNA-seq(page 12-13)

Appendix Table S3. Clinical characteristics(page 14)

Appendix Table S4. Clinical information of Discovery Cohort(page 15)

Appendix Table S5. Clinical information of Plasma validation cohort and Tissue validation cohort(page 16)

Appendix Table S6. The level of PE(18:0/18:1) in cell assay(page 17)

Appendix Table S7. KEGG pathways involved by PE(18:0.18:1)(page 18)

Appendix Table S8. Clinical information of TCGA-LUAD(page 19-23)

Appendix Table S9. Clinical information of CPTAC-LUAD(page 24-26)

Appendix Table S10. The level of PE(18:0/18:1) and enrichment score of hsa00564 in cell assay(page 27).

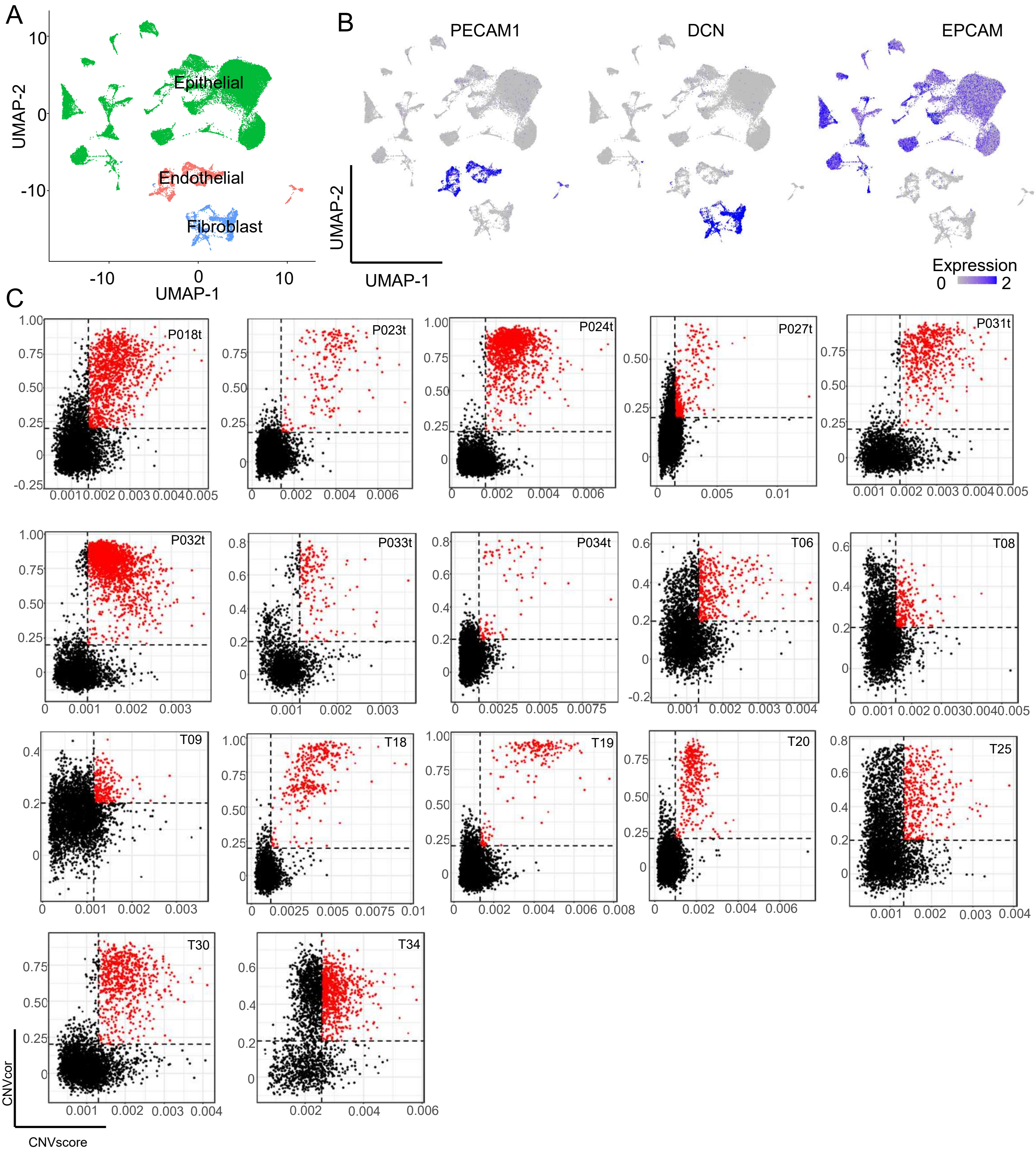

Appendix Figure S1. Malignant epithelial cells identified in ScRNA-seq. (A) The UMAP of 44480 nonimmune cells identified by scRNA-seq. (B) Canonical markers of different cell types used to cell subpopulations in UMAP plot. (C) Scatter plots showing the CNVcor (y axis) versus the CNVscore (x axis) for malignant (red) and non-malignant (black) cells for each samples. Vertical dashed lines indicate the mean+sd value of the CNVscore of each sample immune cells. Horizontal line indicates a CNVcor value of 0.2.

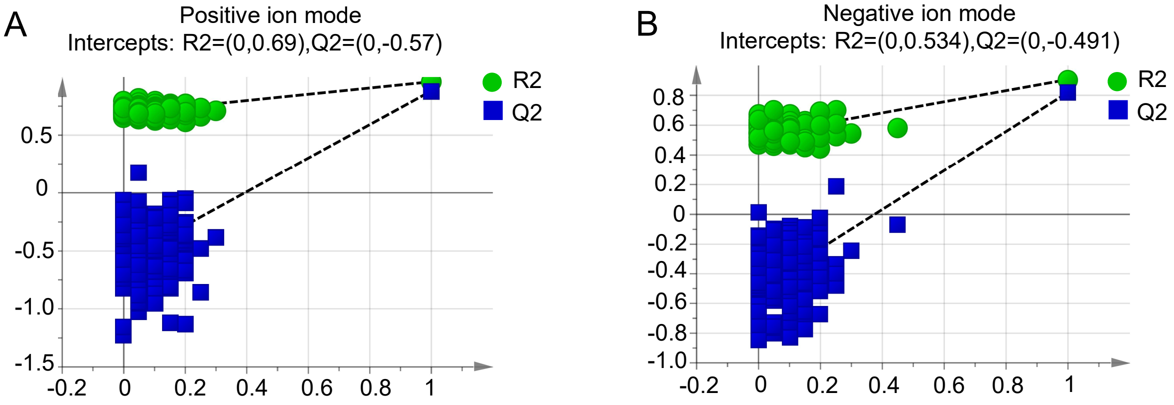

Appendix Figure S2. Permutation test plot for the OPLS-DA model at positive ion mode (A) and negative ion mode (B). The number of permutations is 200. The R2 and Q2 values of the permutated model are on the left-hand side of the panel, corresponding to y-axis intercepts. n=90 samples with 60 LUADs and 30 Healthy control.

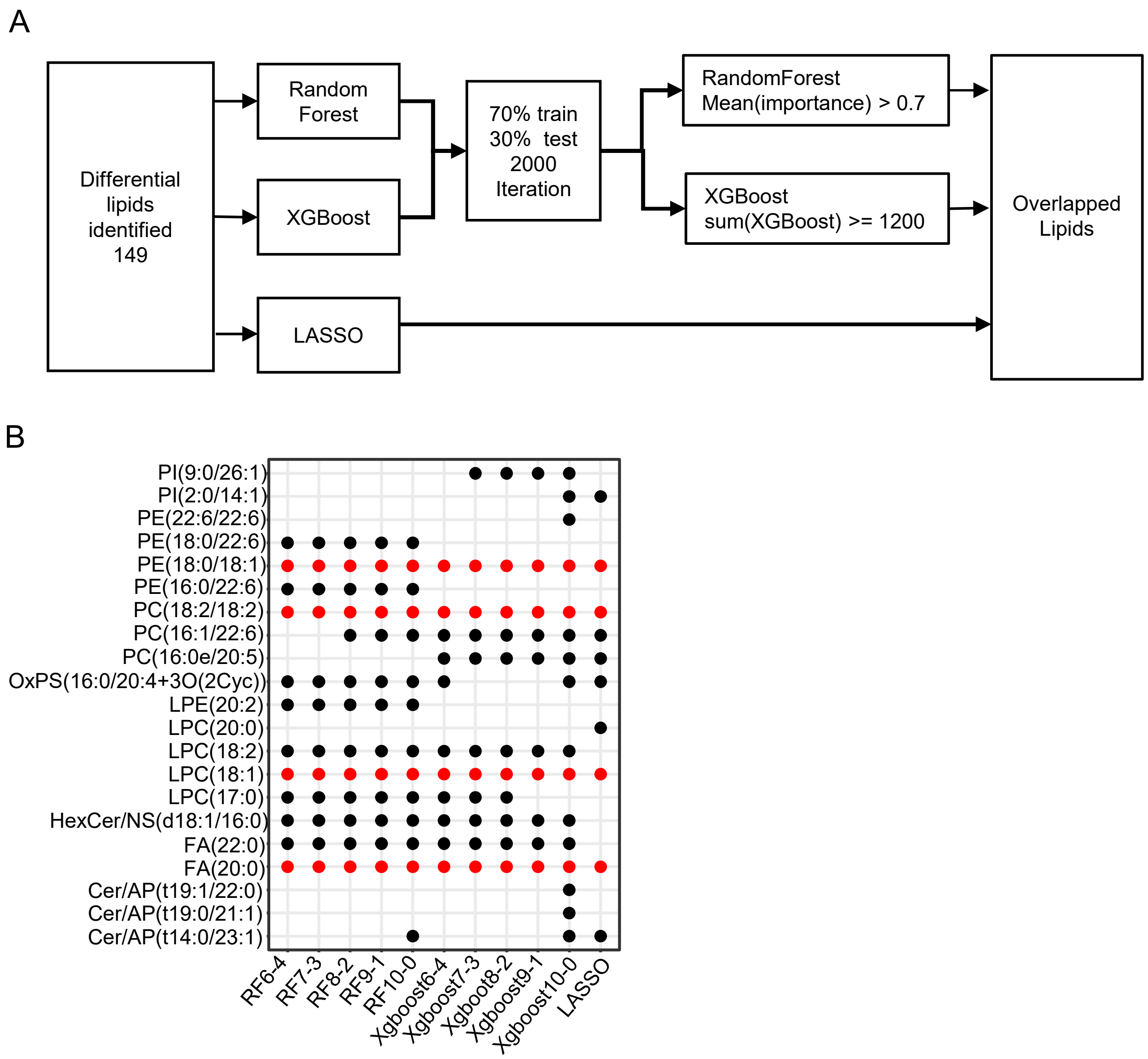

Appendix Figure S3. The workflow of identifying signature lipids from untargeted lipidomics data of plasma. (A)The workflow of identifying signature lipids from untargeted lipidomics data of plasma. (B) Results of screening lipid features with different methods and different ratios of training set/test set in negative ion mode. Each point indicates the lipid marker (y-axis) screened with the corresponding method (x-axis). Red dots indicate 4 lipid markers. n = 2000 iterations. RF6-4: Random Forest applied on training and test sets at ratio of 6:4. RF7-3: Random Forest applied on training and test sets at ratio of 7:3. RF8-2: Random Forest applied on training and test sets at ratio of 8:2. RF9-1: Random Forest applied on training and test sets at ratio of 9:1. RF10-0: Random Forest applied on training and test sets at ratio of 10:0. Xgboost6-4: XGBOOST applied on training and test sets at ratio of 6:4. Xgboost7-3: XGBOOST applied on training and test sets at ratio of 7:3. Xgboost8-2: XGBOOST applied on training and test sets at ratio of 8:2. Xgboost9-1: XGBOOST applied on training and test sets at ratio of 9:1. Xgboost10-0: XGBOOST applied on training and test sets at ratio of 10:0.

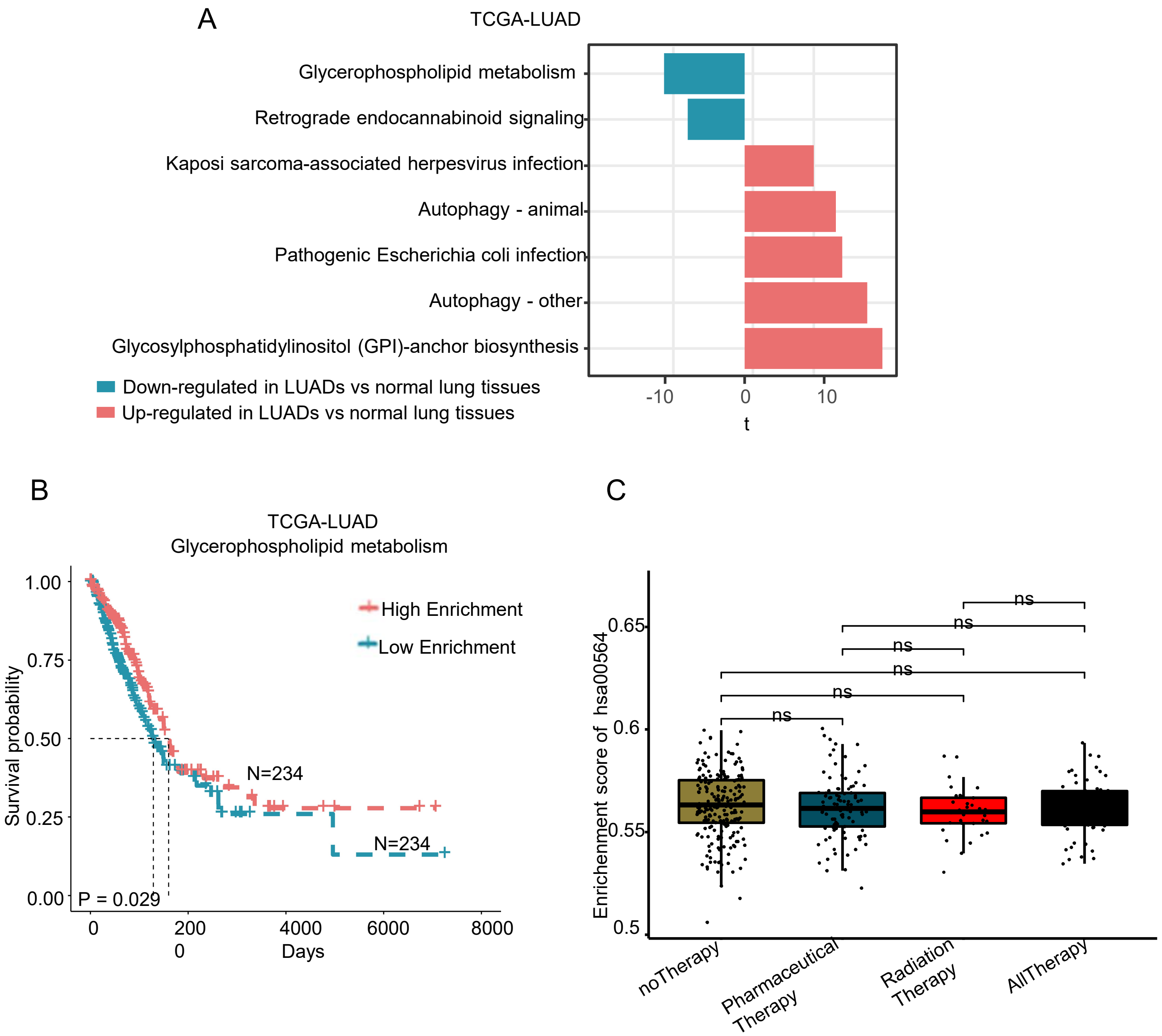

Appendix Figure S4. KEGG pathways associated with PE(18:0/18:1) in TCGA-LUAD. (A) The enrichment status of KEGG pathways involving PE(18:0/18:1). n= 487 tumor tissues from TCGA-LUAD and 120 Healthy lung tissues. (B)Kaplan-Meier curves show the overall survival of LUAD patients with high or low enrichment of glycerophospholipid metabolism in TCGA-LUAD. Red is LUADs with high enrichment score(>median) of glycerophospholipid metabolism. Blue is LUADs with low enrichment score(≤median) of glycerophospholipid metabolism. P value was calculated by Log-rank test. (C)The comparison of enrichenment score of hsa00564(Glycerophospholipid metabolism) pathway between LUADs with different therapy. ns represents Pvalue from Mann Whitney U test > 0.1.

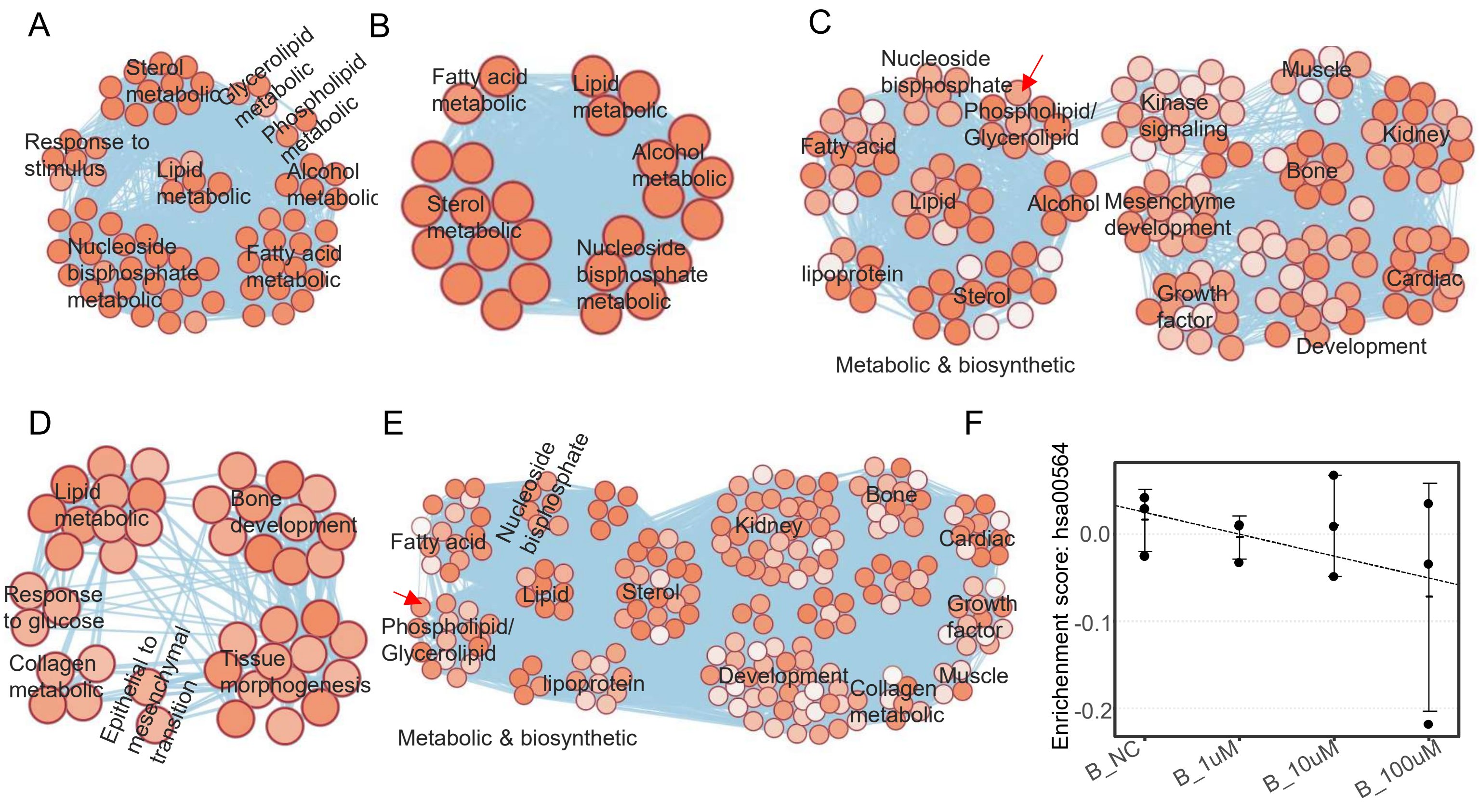

Appendix Figure S5. Enrichment map network of statistically significant GO categories with different concentrations PE(18:0/18:1). Nodes are GO categories and lines their connectivity. A. Compared with the control(0 μmol/L), the pathways down-regulated in PE 10μmol/L. B. Compared with the PE 1μmol/L, the pathways down-regulated in PE 10μmol/L. C. Compared with the control, the pathways down-regulated in PE 100μmol/L. D. Compared with the PE 10μmol/L, the pathways down-regulated in PE 100μmol/L. E. Compared with the PE 1μmol/L, the pathways down-regulated in PE 100μmol/L. F. The enrichment status of glycerophospholid metabolism pathways with different concentrations PE(18:0/18:1). with 3 independent repetitions. Red arrows indicate of glycerophospholid metabolism pathway.

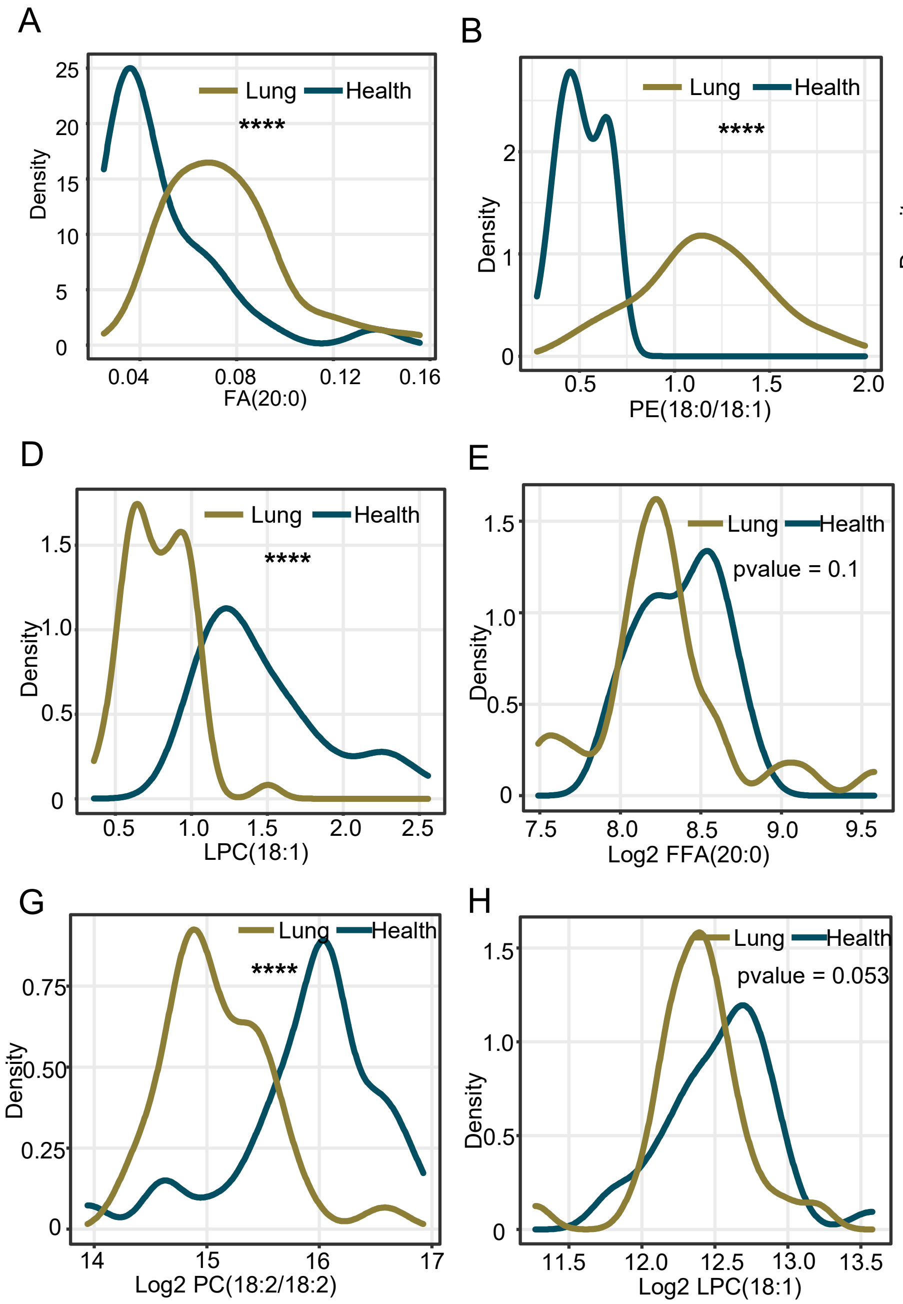

Appendix Figure S6. The distribution of 4 lipid markers. (A-D). The distribution of 4 lipid markers in Discovery cohort.N=60 Lung and 30 Health. **** represents P value from Mann Whitney U test < 0.0001. (E-H). The distribution of 4 lipid marker in plasma validation cohort.N=30 Lung and 30 Health. Pvalue was calculated by from Mann Whitney U test and **** represents P value < 0.0001.

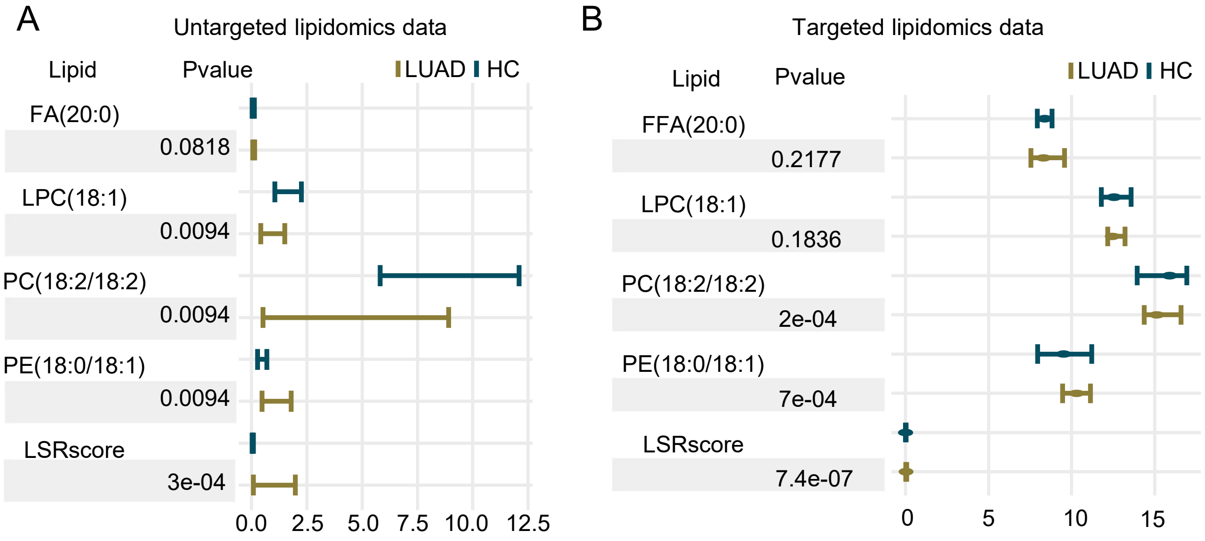

Appendix Figure S7. The robustness of the 4 lipid markers to age. The comparison of 4 lipid markers concentration between age-matched HC and LUAD in Discovery Cohort(A)(LUAD:n=5;HC:n=12) and plasma validation cohort(B)(LUAD:n=15;HC:n=30). The P value calculated by Mann Whitney U test. The level of 4 lipid markers in Targeted lipidomics data was log2 processed for visualizing.

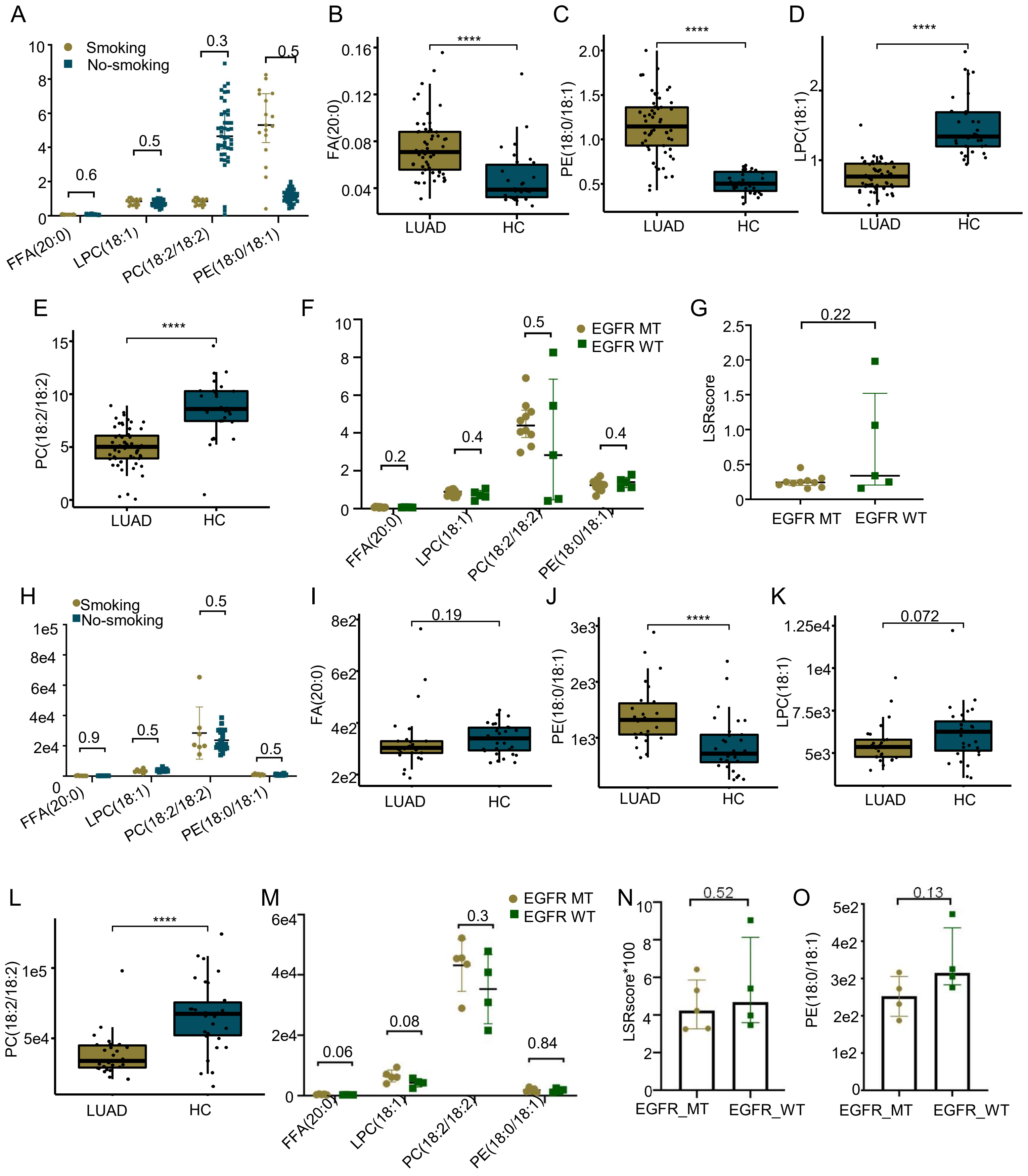

Appendix Figure S8. Analysis of clinical information and 4 lipid markers between LUADs and HC in discovery cohort and plasma validation cohort. (A,H)The comparison of lipid concentration between LUADs with smoking and LUADs without smoking for 4 lipid markers in discovery cohort(A)(n = 17 for Smoking and n=43 for No-smoking) and plasma validation cohort(H)(n = 7 for Smoking and n= 23 for No-smoking) . Pvalue were calculated by Student's t-tests. (B-E) The comparison of 4 lipid markers level between LUADs and HC after removing LUADs with other lung diseases in the discovery cohort(n=57 for LUAD and n=30 for HC). **** represents P value from Mann Whitney U test < 0.0001. (I-L) The comparison of 4 lipid markers level between LUADs and HC after removing LUADs with other lung diseases in plasma validation cohort(n= 26 for LUAD and n=30 for HC). P value was calculated by Mann Whitney U test and **** represents P value< 0.0001. (F,M)The level of lipid markers between LUADs with EGFR mutations and LUADs without EFGR mutations in discovery cohort(F, n=10 for EGFR MT and n=5 for EGFR WT) and plasma validation cohort(M,n=5 for EGFR MT and n=4 for EGFR WT). Pvalue were calculated by Student's t-tests. (G,N)The comparison of LSRscore between LUADs with different EGFR mutatant status in the discovery cohort(G, n=10 for EGFR MT and n=5 for EGFR WT) and plasma validation cohort(N). (O) The level of PE(18:0/18:1) between LUADs with different EGFR mutation in the tissue validation cohort. P values were calculated by Student's t-tests in G,N,O.

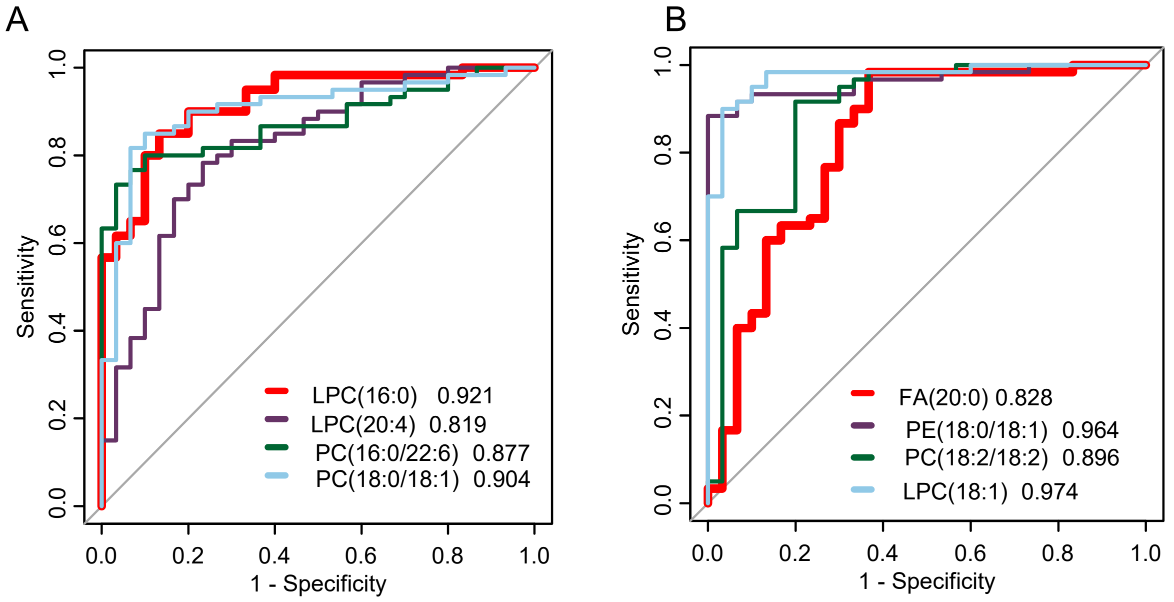

Appendix Figure S9. ROC curves of 4 lipid markers in the discovery cohort. (A). Of the 9 lipid markers identified by Wang et al, 4 were also detected in the plasma samples in our Discovery LUAD cohort.(B) 4 lipid signatures in this study.

Appendix Table S1: Clinical information of patients used for single-cell RNA data

Sample ID	Age (yr)	Sex	Smoking Status	Stages	tissue_type
8 healthy lung sample					
Donor 1	63	F	Never	–	–
Donor 2	55	M	Former	–	–
Donor 3	29	F	Never	–	–
Donor 4	57	F	Never	–	–
Donor 5	49	F	Active	–	–
Donor 6	22	F	Never	–	–
Donor 7	47	F	Active	–	–
Donor 8	21	M	Never	–	–
Kim N et.al (2020)					
LUNG_N01	–	–	Never	IA	Normal
LUNG_N06	–	–	Ex	IA	Normal
LUNG_N08	–	–	Never	IB	Normal
LUNG_N09	–	–	Ex	IIA	Normal
LUNG_N18	–	–	Ex	IA	Normal
LUNG_N19	–	–	Cur	IA	Normal
LUNG_N20	–	–	Cur	IA	Normal
LUNG_N30	–	–	Never	IA	Normal
LUNG_N34	–	–	Never	IA3	Normal
LUNG_T06	–	–	Ex	IA	Tumor
LUNG_T08	–	–	Never	IB	Tumor
LUNG_T09	–	–	Ex	IIA	Tumor
LUNG_T18	–	–	Ex	IA	Tumor
LUNG_T19	–	–	Cur	IA	Tumor
LUNG_T20	–	–	Cur	IA	Tumor
LUNG_T25	–	–	Ex	IA	Tumor
LUNG_T30	–	–	Never	IA	Tumor
LUNG_T34	–	–	Never	IA3	Tumor
Bischoff P et.al(2021)					
p018t	58	F	–	T1aN0	Tumor
p023t	62	F	–	T1cN0	Tumor
p024t	63	F	–	T1bN0	Tumor
p027t	49	M	–	T2aN0	Tumor
p031t	77	M	–	T2N0	Tumor
p032t	83	F	–	T2N0	Tumor
p033t	64	F	–	T3N0	Tumor
p034t	63	M	–	T2bN0	Tumor
p018n	58	F	–	T1aN0	Normal
p027n	49	M	–	T2aN0	Normal
p028n	77	M	–	T2bN2	Normal
p029n	59	F	–	T1bN1b	Normal
p031n	77	M	–	T2N0	Normal
p032n	83	F	–	T2N0	Normal
p033n	64	F	–	T3N0	Normal
p034n	63	M	–	T2bN0	Normal

Appendix Table S2.GO terms down-regulated in tumor maliganat epithelial vs nonmalignant epithelial cells in ScRNA-seq

Function	ID	Description	P-value	Q-value	geneID	Count
Nucleoside bisphosphate metabolic	GO:0033865	nucleoside bisphosphate metabolic process	0.000541248	0.021	ACSL1,HMGCS1,HACD1,MVD,ACSS2,HMGCR,ACAT1,ELOVL5,ACSL4,HSD17B4,HSD17B12	11
	GO:0033875	ribonucleoside bisphosphate metabolic process	0.000541248	0.021	ACSL1,HMGCS1,HACD1,MVD,ACSS2,HMGCR,ACAT1,ELOVL5,ACSL4,HSD17B4,HSD17B12	11
	GO:0034032	purine nucleoside bisphosphate metabolic process	0.000541248	0.021	ACSL1,HMGCS1,HACD1,MVD,ACSS2,HMGCR,ACAT1,ELOVL5,ACSL4,HSD17B4,HSD17B12	11
	GO:0033866	nucleoside bisphosphate biosynthetic process	0.000909391	0.03	ACSL1,HACD1,ACSS2,ACAT1,ELOVL5,ACSL4,HSD17B12	7
	GO:0034030	ribonucleoside bisphosphate biosynthetic process	0.000909391	0.03	ACSL1,HACD1,ACSS2,ACAT1,ELOVL5,ACSL4,HSD17B12	7
	GO:0034033	purine nucleoside bisphosphate biosynthetic process	0.000909391	0.03	ACSL1,HACD1,ACSS2,ACAT1,ELOVL5,ACSL4,HSD17B12	7
	GO:0009150	purine ribonucleotide metabolic process	0.001370105	0.04	ACSL1,HMGCS1,NDUFB9,AK1,HACD1,LRRK2,NDUFB1,NUPR1,MVD,ACSS2,HMGCR,PDE4D,PID1,ACAT1,ELOVL5,NDUFA1,ACSL4,NDUFA7,TJP2,ARL2,NDUFA3,PFKFB2,HSD17B4,HSD17B12	24
	GO:0035384	thioester biosynthetic process	0.000238634	0.013	ACSL1,HACD1,ACSS2,ACAT1,ELOVL5,ACSL4,HSD17B12	7
Phospholipid metabolic	GO:0006644	phospholipid metabolic process	4.26E-09	0	MFSD2A,FDPS,DBI,LPCAT1,HMGCS1,LPL,FABP5,PIP5K1B,FADS1,IDI1,PLA2G1B,CHP1,PLA2G4F,ABCA3,MVD,LDLR,APOC1,FDFT1,PCSK9,GPX4,CLN3,ALOX15B,PCYT2,AGPAT2,SOCS2,CHPT1,DPM3,SH3YL1,PTPMT1,IMPA2,PLA2G10,SGMS2,SOCS3	33
	GO:0046474	glycerophospholipid biosynthetic process	0.002051631	0.05	MFSD2A,LPCAT1,FABP5,PIP5K1B,CLN3,PCYT2,AGPAT2,SOCS2,CHPT1,DPM3,SH3YL1,PTPMT1,IMPA2,SOCS3	14
	GO:1903725	regulation of phospholipid metabolic process	0.000401837	0.019	MFSD2A,LPCAT1,CHP1,ABCA3,LDLR,APOC1	6
	GO:0006650	glycerophospholipid metabolic process	7.80E-05	0.006	MFSD2A,DBI,LPCAT1,FABP5,PIP5K1B,PLA2G1B,PLA2G4F,ABCA3,LDLR,APOC1,CLN3,PCYT2,AGPAT2,SOCS2,CHPT1,DPM3,SH3YL1,PTPMT1,IMPA2,PLA2G10,SOCS3	21
	GO:0008654	phospholipid biosynthetic process	8.14E-06	0.001	MFSD2A,FDPS,LPCAT1,HMGCS1,FABP5,PIP5K1B,FADS1,IDI1,CHP1,MVD,CLN3,PCYT2,AGPAT2,SOCS2,CHPT1,DPM3,SH3YL1,PTPMT1,IMPA2,SGMS2,SOCS3	21
	GO:0046486	glycerolipid metabolic process	3.05E-06	0	MFSD2A,DBI,ACSL1,LPCAT1,INSIG1,LPL,FABP5,PIP5K1B,PLA2G1B,PLA2G4F,ABCA3,LDLR,APOC1,GPX1,PCSK9,CAT,CLN3,PCYT2,AGPAT2,SOCS2,CAV1,CHPT1,DPM3,SH3YL1,PTPMT1,IMPA2,PLA2G10,SOCS3	28
	GO:0045017	glycerolipid biosynthetic process	0.000573123	0.022	MFSD2A,ACSL1,LPCAT1,LPL,FABP5,PIP5K1B,LDLR,CLN3,PCYT2,AGPAT2,SOCS2,CHPT1,DPM3,SH3YL1,PTPMT1,IMPA2,SOCS3	17
	GO:0150172	regulation of phosphatidylcholine metabolic process	3.44E-06	0	MFSD2A,LPCAT1,ABCA3,LDLR,APOC1	5
	GO:0046471	phosphatidylglycerol metabolic process	0.000342725	0.017	LPCAT1,PLA2G1B,PLA2G4F,ABCA3,PTPMT1,PLA2G10	6
	GO:0046470	phosphatidylcholine metabolic process	4.19E-05	0.003	MFSD2A,DBI,LPCAT1,FABP5,PLA2G1B,ABCA3,LDLR,APOC1,CHPT1,PLA2G10	10
	GO:0043129	surfactant homeostasis	7.20E-05	0.006	LPCAT1,SFTPD,CTSH,ABCA3,EPAS1	5
	GO:0140962	multicellular organismal-level chemical homeostasis	0.000816706	0.028	LPCAT1,SFTPD,CTSH,ABCA3,AQP4,EPAS1,SCNN1A	7
	GO:0015748	organophosphate ester transport	0.000241624	0.013	MFSD2A,DBI,SLC25A6,SLC25A5,NPC2,ABCA3,LDLR,APOC1,TMEM41B,ATP8A1,SLC25A4,PLA2G10,SLC25A24	13
Lipid metabolic/ localization	GO:1905954	positive regulation of lipid localization	0.00025862	0.014	DBI,ACSL1,CD36,LPL,ABCA3,NFKBIA,CAV1,ATP8A1,PLIN2,DAB2,PLA2G10	11
	GO:0019915	lipid storage	0.00058223	0.022	CD36,LPL,SQLE,NFKBIA,STARD4,ALKBH7,CAV1,PLIN2,PLA2G10	9
	GO:1905952	regulation of lipid localization	0.001591985	0.043	DBI,ACSL1,CD36,LPL,ABCA3,APOC1,PCSK9,NFKBIA,CAV1,ATP8A1,PLIN2,DAB2,PLA2G10	13
	GO:0019216	regulation of lipid metabolic process	0.00139561	0.04	MFSD2A,ACADL,LPCAT1,INSIG1,MID1IP1,FABP5,DHCR7,NPC2,CHP1,ABCA3,LDLR,APOC1,ASAH1,STARD4,ELOVL5,DNAJC15,CAV1,TRIB3,DAB2,BMP2	20
	GO:0006869	lipid transport	0.000656439	0.024	MFSD2A,DBI,ACSL1,CD36,FABP5,SFTPA1,PLA2G1B,NPC2,PLA2G4F,ABCA3,LDLR,APOC1,PCSK9,TMEM41B,NPC1,NFKBIA,CLN3,STARD4,SLC51B,CAV1,ATP8A1,PLIN2,STARD3NL,DAB2,PLA2G10	25
	GO:0042180	cellular ketone metabolic process	0.00032346	0.016	MFSD2A,ACADL,INSIG1,MID1IP1,FABP5,APOC1,EDNRB,CLN3,STARD4,ELOVL5,CAV1,TRIB3,DAB2,BMP2,KDSR,NMT1	16
	GO:0010565	regulation of cellular ketone metabolic process	8.95E-05	0.006	MFSD2A,ACADL,INSIG1,MID1IP1,FABP5,APOC1,CLN3,STARD4,ELOVL5,CAV1,TRIB3,DAB2,BMP2	13
	GO:0010743	regulation of macrophage derived foam cell differentiation	0.001667786	0.043	CD36,LPL,NFKBIA,ALOX15B,PLA2G10	5
	GO:0010744	positive regulation of macrophage derived foam cell differentiation	0.001567133	0.043	CD36,LPL,ALOX15B,PLA2G10	4
	GO:0062012	regulation of small molecule metabolic process	0.000875711	0.03	MFSD2A,ACADL,INSIG1,MID1IP1,FABP5,DHCR7,NUPR1,LDLR,APOC1,PID1,NNMT,CLN3,STARD4,ELOVL5,CAV1,TRIB3,ARL2,PFKFB2,DAB2,BMP2	20
	GO:0006641	triglyceride metabolic process	0.000422626	0.019	MFSD2A,ACSL1,INSIG1,LPL,LDLR,APOC1,GPX1,PCSK9,CAT,CAV1	10
	GO:0010876	lipid localization	0.000282843	0.014	MFSD2A,DBI,ACSL1,CD36,LPL,FABP5,SQLE,SFTPA1,PLA2G1B,NPC2,PLA2G4F,ABCA3,LDLR,APOC1,PCSK9,TMEM41B,NPC1,NFKBIA,CLN3,STARD4,SLC51B,ALKBH7,CAV1,ATP8A1,PLIN2,STARD3NL,DAB2,PLA2G10	28
	GO:0046890	regulation of lipid biosynthetic process	1.16E-05	0.001	MFSD2A,ACADL,LPCAT1,INSIG1,MID1IP1,FABP5,DHCR7,CHP1,ABCA3,LDLR,APOC1,ASAH1,STARD4,ELOVL5,TRIB3,DAB2,BMP2	17
Sterol metabolic	GO:0016125	sterol metabolic process	6.37E-12	0	DHCR24,FDPS,ACADL,MSMO1,HMGCS1,INSIG1,EBP,SQLE,DHCR7,IDI1,NPC2,MVD,LDLR,APOC1,LSS,HMGCR,FDFT1,CYP51A1,PCSK9,NPC1,CAT,STARD4,TM7SF2,SC5D	24
	GO:0055092	sterol homeostasis	0.001719529	0.043	INSIG1,LPL,NPC2,TMEM97,LDLR,PCSK9,NPC1,CAV1,PLA2G10	9
	GO:0032367	intracellular cholesterol transport	0.001667786	0.043	NPC2,LDLR,PCSK9,NPC1,STARD4	5
	GO:0042632	cholesterol homeostasis	0.001602808	0.043	INSIG1,LPL,NPC2,TMEM97,LDLR,PCSK9,NPC1,CAV1,PLA2G10	9
	GO:0015918	sterol transport	0.000427864	0.019	CD36,NPC2,ABCA3,LDLR,APOC1,PCSK9,NPC1,NFKBIA,STARD4,CAV1,STARD3NL,PLA2G10	12
	GO:0030301	cholesterol transport	0.000158069	0.01	CD36,NPC2,ABCA3,LDLR,APOC1,PCSK9,NPC1,NFKBIA,STARD4,CAV1,STARD3NL,PLA2G10	12
	GO:0016126	sterol biosynthetic process	9.20E-12	0	DHCR24,FDPS,MSMO1,HMGCS1,INSIG1,EBP,SQLE,DHCR7,IDI1,MVD,LSS,HMGCR,FDFT1,CYP51A1,TM7SF2,SC5D	16
	GO:0006695	cholesterol biosynthetic process	1.79E-11	0	DHCR24,FDPS,MSMO1,HMGCS1,INSIG1,EBP,DHCR7,IDI1,MVD,LSS,HMGCR,FDFT1,CYP51A1,TM7SF2,SC5D	15
	GO:0008203	cholesterol metabolic process	6.55E-13	0	DHCR24,FDPS,ACADL,MSMO1,HMGCS1,INSIG1,EBP,SQLE,DHCR7,IDI1,NPC2,MVD,LDLR,APOC1,LSS,HMGCR,FDFT1,CYP51A1,PCSK9,NPC1,CAT,STARD4,TM7SF2,SC5D	24
	GO:0006694	steroid biosynthetic process	2.89E-09	0	DHCR24,FDPS,MSMO1,HMGCS1,INSIG1,EBP,SQLE,DHCR7,IDI1,MVD,LSS,HMGCR,ASAH1,FDFT1,CYP51A1,STARD4,DAB2,BMP2,TM7SF2,HINT2,SC5D,HSD17B12	22
	GO:0008202	steroid metabolic process	1.60E-09	0	DHCR24,FDPS,ACADL,MSMO1,HMGCS1,INSIG1,EBP,SQLE,DHCR7,IDI1,NPC2,MVD,LDLR,APOC1,LSS,HMGCR,EDNRB,ASAH1,FDFT1,CYP51A1,PCSK9,NPC1,CAT,STARD4,DAB2,HSD17B4,BMP2,TM7SF2,HINT2,SC5D,HSD17B12	31
	GO:1902653	secondary alcohol biosynthetic process	1.79E-11	0	DHCR24,FDPS,MSMO1,HMGCS1,INSIG1,EBP,DHCR7,IDI1,MVD,LSS,HMGCR,FDFT1,CYP51A1,TM7SF2,SC5D	15
	GO:0046165	alcohol biosynthetic process	3.61E-11	0	DHCR24,FDPS,MSMO1,HMGCS1,INSIG1,EBP,DHCR7,IDI1,SPTSSA,MVD,LSS,HMGCR,ASAH1,FDFT1,CYP51A1,PCBD1,QDPR,DAB2,BMP2,IMPA2,TM7SF2,SC5D	22
	GO:0006066	alcohol metabolic process	3.70E-11	0	DHCR24,FDPS,ACADL,MSMO1,HMGCS1,INSIG1,EBP,SDR16C5,SQLE,DHCR7,IDI1,SPTSSA,NPC2,MVD,ACSS2,LDLR,APOC1,LSS,HMGCR,EDNRB,ASAH1,FDFT1,CYP51A1,PCSK9,NPC1,CAT,STARD4,PCBD1,DPM3,RDH11,QDPR,DAB2,BMP2,IMPA2,TM7SF2,SC5D	36
	GO:1902652	secondary alcohol metabolic process	3.08E-12	0	DHCR24,FDPS,ACADL,MSMO1,HMGCS1,INSIG1,EBP,SQLE,DHCR7,IDI1,NPC2,MVD,LDLR,APOC1,LSS,HMGCR,FDFT1,CYP51A1,PCSK9,NPC1,CAT,STARD4,TM7SF2,SC5D	24
	GO:0006720	isoprenoid metabolic process	0.00043764	0.019	FDPS,HMGCS1,SDR16C5,IDI1,NPC2,MVD,LSS,HMGCR,FDFT1,DPM3,RDH11	11
	GO:0008299	isoprenoid biosynthetic process	0.000140822	0.009	FDPS,HMGCS1,IDI1,MVD,LSS,HMGCR	6
	GO:1901617	organic hydroxy compound biosynthetic process	3.22E-09	0	DHCR24,FDPS,MSMO1,HMGCS1,INSIG1,EBP,SQLE,DHCR7,IDI1,SPTSSA,MVD,LSS,HMGCR,ASAH1,FDFT1,CYP51A1,DDT,ALOX15B,STARD4,PCBD1,QDPR,DAB2,BMP2,IMPA2,TM7SF2,SC5D	26
Fatty acid metabolic	GO:1901570	fatty acid derivative biosynthetic process	0.000517683	0.021	ACSL1,HACD1,ALOX15B,ACAT1,ELOVL5,ACSL4,HSD17B12	7
	GO:0070542	response to fatty acid	0.001652819	0.043	ACSL1,CD36,LPL,SCD,LDLR,PID1,CAT	7
	GO:1901568	fatty acid derivative metabolic process	0.001221411	0.037	ACSL1,HACD1,ALOX15B,ACAT1,ELOVL5,ACSL4,HSD17B4,HSD17B12	8
	GO:0033559	unsaturated fatty acid metabolic process	0.001182544	0.037	FABP5,FADS2,FADS1,SCD,PLA2G4F,GPX1,GPX4,ALOX15B,ELOVL5,PLA2G10	10
	GO:0019217	regulation of fatty acid metabolic process	0.001197752	0.037	MFSD2A,ACADL,INSIG1,MID1IP1,FABP5,APOC1,ELOVL5,CAV1,TRIB3	9
	GO:0001676	long-chain fatty acid metabolic process	0.000897914	0.03	ACADL,ACSL1,FADS2,FADS1,GPX1,GPX4,ALOX15B,ELOVL5,ACSL4,PLA2G10	10
	GO:0015909	long-chain fatty acid transport	0.000531787	0.021	MFSD2A,ACSL1,CD36,FABP5,PLA2G1B,PLA2G4F,PLIN2,PLA2G10	8
	GO:0042304	regulation of fatty acid biosynthetic process	0.000405177	0.019	ACADL,INSIG1,MID1IP1,FABP5,APOC1,ELOVL5,TRIB3	7
	GO:0006636	unsaturated fatty acid biosynthetic process	7.94E-05	0.006	FABP5,FADS2,FADS1,SCD,PLA2G4F,ALOX15B,ELOVL5,PLA2G10	8
	GO:0006633	fatty acid biosynthetic process	9.70E-08	0	ACADL,INSIG1,MID1IP1,LPL,FABP5,FADS2,FADS1,HACD1,SCD,PLA2G1B,PLA2G4F,FASN,APOC1,GPX4,ALOX15B,ELOVL5,TRIB3,PLA2G10,HSD17B12	19
	GO:0006631	fatty acid metabolic process	1.11E-09	0	MFSD2A,DBI,ACADL,MSMO1,ACSL1,ACAT2,CD36,INSIG1,MID1IP1,LPL,FABP5,FADS2,FADS1,HACD1,SCD,PLA2G1B,PLA2G4F,FASN,APOC1,GPX1,ASAH1,GPX4,ALOX15B,ACAT1,ELOVL5,ALKBH7,ACSL4,CAV1,TRIB3,ECH1,CYP4B1,HSD17B4,ECHDC1,PLA2G10,HSD17B12	35
	GO:0046949	fatty-acyl-CoA biosynthetic process	7.53E-05	0.006	ACSL1,HACD1,ACAT1,ELOVL5,ACSL4,HSD17B12	6
	GO:0035337	fatty-acyl-CoA metabolic process	9.58E-05	0.007	ACSL1,HACD1,ACAT1,ELOVL5,ACSL4,HSD17B4,HSD17B12	7
	GO:0035338	long-chain fatty-acyl-CoA biosynthetic process	0.000129223	0.009	ACSL1,HACD1,ELOVL5,ACSL4,HSD17B12	5
	GO:0072330	monocarboxylic acid biosynthetic process	3.02E-06	0	ACADL,INSIG1,MID1IP1,LPL,FABP5,FADS2,FADS1,HACD1,SCD,PLA2G1B,PLA2G4F,FASN,APOC1,GPX4,ALOX15B,STARD4,ELOVL5,TRIB3,PLA2G10,HSD17B12	20
	GO:0046394	carboxylic acid biosynthetic process	4.56E-06	0	ACADL,INSIG1,MID1IP1,LPL,FABP5,FADS2,FADS1,HACD1,SCD,PLA2G1B,PLA2G4F,FASN,APOC1,PHGDH,GPX4,CLN3,ALOX15B,STARD4,SLC38A1,ELOVL5,PCBD1,TRIB3,SHMT2,PLA2G10,HSD17B12	25
	GO:0036109	alpha-linolenic acid metabolic process	0.002137072	0.05	FADS2,FADS1,ELOVL5	3
	GO:0016053	organic acid biosynthetic process	5.35E-06	0	ACADL,INSIG1,MID1IP1,LPL,FABP5,FADS2,FADS1,HACD1,SCD,PLA2G1B,PLA2G4F,FASN,APOC1,PHGDH,GPX4,CLN3,ALOX15B,STARD4,SLC38A1,ELOVL5,PCBD1,TRIB3,SHMT2,PLA2G10,HSD17B12	25
	GO:0006637	acyl-CoA metabolic process	0.000255475	0.014	ACSL1,HMGCS1,HACD1,MVD,ACSS2,ACAT1,ELOVL5,ACSL4,HSD17B4,HSD17B12	10
	GO:0035336	long-chain fatty-acyl-CoA metabolic process	0.000515338	0.021	ACSL1,HACD1,ELOVL5,ACSL4,HSD17B12	5
	GO:0071616	acyl-CoA biosynthetic process	0.000238634	0.013	ACSL1,HACD1,ACSS2,ACAT1,ELOVL5,ACSL4,HSD17B12	7
	GO:0006081	cellular aldehyde metabolic process	0.000587336	0.022	ALPL,SDR16C5,EDNRB,ESD,RDH11,DAB2,BMP2,HYI	8
	GO:0120254	olefinic compound metabolic process	0.001415347	0.04	SDR16C5,FADS2,FADS1,GPX1,EDNRB,GPX4,ALOX15B,ELOVL5,RDH11,DAB2,BMP2,PLA2G10	12
	GO:0035383	thioester metabolic process	0.000255475	0.014	ACSL1,HMGCS1,HACD1,MVD,ACSS2,ACAT1,ELOVL5,ACSL4,HSD17B4,HSD17B12	10
Protein catabolic	GO:0045022	early endosome to late endosome transport	0.001210134	0.037	SNX3,RILP,EMP2,DAB2,CHMP3,EZR	6
	GO:0032387	negative regulation of intracellular transport	0.001504061	0.042	ERLEC1,CD36,INSIG1,LRRK2,CHP1,PCSK9,SNX3	7
	GO:1903828	negative regulation of protein localization	0.002051631	0.05	ERLEC1,CLDN18,CD36,INSIG1,LRRK2,CHP1,HMGCR,CYP51A1,PID1,ERP29,SNX3,NEDD4L,NDUFAF2,DAB2	14
	GO:1904951	positive regulation of establishment of protein localization	0.000698665	0.025	TTN,SERP1,TOMM7,PLA2G1B,CHP1,OAZ1,CLN3,UBL5,SLC51B,ACSL4,PFKFB2,CD38,VAMP2,SAR1A,STOM,IER3IP1,ATP2C1,PRKCZ,EZR,NMT1	20
	GO:0045732	positive regulation of protein catabolic process	0.001962778	0.048	LPCAT1,LRRK2,NUPR1,OAZ1,LDLR,EGLN2,GABARAP,PCSK9,CAV1,TRIB3,RNF128,RILP,DAB2,EZR	14
	GO:0051222	positive regulation of protein transport	0.000369087	0.018	TTN,SERP1,TOMM7,PLA2G1B,CHP1,OAZ1,CLN3,UBL5,SLC51B,ACSL4,PFKFB2,CD38,VAMP2,SAR1A,STOM,IER3IP1,ATP2C1,PRKCZ,EZR,NMT1	20
	GO:1903649	regulation of cytoplasmic transport	0.001052328	0.034	LRRK2,SNX3,DAB2,CHMP3,EZR	5
	GO:0033157	regulation of intracellular protein transport	0.001380682	0.04	ERLEC1,TOMM7,CD36,INSIG1,LRRK2,CHP1,OAZ1,UBL5,SLC51B,NUP58,PTPN1,VAMP2,SAR1A,STOM,NMT1	15
	GO:0032386	regulation of intracellular transport	0.000908689	0.03	ERLEC1,TOMM7,CD36,INSIG1,LRRK2,CHP1,OAZ1,PCSK9,UBL5,SLC51B,SNX3,NUP58,PTPN1,VAMP2,SAR1A,STOM,DAB2,CHMP3,EZR,NMT1	20
	GO:0042176	regulation of protein catabolic process	0.001063843	0.034	RPL11,LAMP3,LPCAT1,EEF1A1,LRRK2,NUPR1,OAZ1,LDLR,EGLN2,GPX1,HMGCR,GABARAP,CYP51A1,PCSK9,CAV1,TRIB3,SNX3,RNF128,RILP,DAB2,EZR	21
	GO:2000641	regulation of early endosome to late endosome transport	0.001567133	0.043	SNX3,DAB2,CHMP3,EZR	4
	GO:0098927	vesicle-mediated transport between endosomal compartments	0.00171762	0.043	SNX3,RILP,EMP2,DAB2,CHMP3,EZR	6
	GO:0032527	protein exit from endoplasmic reticulum	0.001918263	0.048	ERLEC1,INSIG1,SLC51B,SEC61B,RANGRF,SAR1A	6

ATP synthesis & mitochondrial	GO:0042775	mitochondrial ATP synthesis coupled electron transport	4.22E-12	0	COX7C,UQCRQ,COX7A2,NDUFA4,COX7B,UQCRB,NDUFB9,COX8A,NDUFB1,COX4I1,UQCR11,COX5A,NDUFA1,DNAJC15,NDUFA7,NDUFA3,COX6A1,COX6C,COX7A2L,GHITM	20
	GO:0006123	mitochondrial electron transport, cytochrome c to oxygen	5.03E-10	0	COX7C,COX7A2,NDUFA4,COX7B,COX8A,COX4I1,COX5A,COX6A1,COX6C,COX7A2L	10
	GO:0006120	mitochondrial electron transport, NADH to ubiquinone	0.000458686	0.02	NDUFA4,NDUFB9,NDUFB1,NDUFA1,DNAJC15,NDUFA7,NDUFA3	7
	GO:0033108	mitochondrial respiratory chain complex assembly	0.001602808	0.043	COX17,NDUFB9,NDUFB1,NDUFA1,NDUFA3,COX14,NDUFAF2,CHCHD7,PET100	9
	GO:0022900	electron transport chain	6.50E-10	0	COX7C,UQCRQ,COX7A2,NDUFA4,COX7B,UQCRB,NDUFB9,COX8A,NDUFB1,COX4I1,CYB5A,UQCR11,COX5A,PHGDH,NDUFA1,DNAJC15,NDUFA7,NDUFA3,COX6A1,COX6C,COX7A2L,QDPR,GHITM	23
	GO:0022904	respiratory electron transport chain	1.74E-10	0	COX7C,UQCRQ,COX7A2,NDUFA4,COX7B,UQCRB,NDUFB9,COX8A,NDUFB1,COX4I1,UQCR11,COX5A,NDUFA1,DNAJC15,NDUFA7,NDUFA3,COX6A1,COX6C,COX7A2L,GHITM	20
	GO:0042773	ATP synthesis coupled electron transport	4.22E-12	0	COX7C,UQCRQ,COX7A2,NDUFA4,COX7B,UQCRB,NDUFB9,COX8A,NDUFB1,COX4I1,UQCR11,COX5A,NDUFA1,DNAJC15,NDUFA7,NDUFA3,COX6A1,COX6C,COX7A2L,GHITM	20
	GO:0019646	aerobic electron transport chain	8.28E-12	0	COX7C,UQCRQ,COX7A2,NDUFA4,COX7B,UQCRB,NDUFB9,COX8A,NDUFB1,COX4I1,UQCR11,COX5A,NDUFA1,DNAJC15,NDUFA7,NDUFA3,COX6A1,COX6C,COX7A2L	19
	GO:0009060	aerobic respiration	1.74E-10	0	COX7C,UQCRQ,COX7A2,NDUFA4,COX7B,UQCRB,NDUFB9,COX8A,NDUFB1,NUPR1,COX4I1,UQCR11,COX5A,MRPS36,CAT,NDUFA1,DNAJC15,NDUFA7,ARL2,NDUFA3,COX6A1,COX6C,COX7A2L,GHITM,SHMT2	25
	GO:0006119	oxidative phosphorylation	1.10E-10	0	COX7C,UQCRQ,COX7A2,NDUFA4,COX7B,UQCRB,NDUFB9,COX8A,NDUFB1,NUPR1,COX4I1,UQCR11,COX5A,NDUFA1,DNAJC15,NDUFA7,NDUFA3,COX6A1,COX6C,COX7A2L,GHITM,SHMT2	22
	GO:0045333	cellular respiration	1.56E-08	0	COX7C,UQCRQ,COX7A2,NDUFA4,COX7B,UQCRB,NDUFB9,COX8A,NDUFB1,NUPR1,COX4I1,UQCR11,COX5A,MRPS36,CAT,NDUFA1,DNAJC15,NDUFA7,ARL2,NDUFA3,COX6A1,COX6C,COX7A2L,GHITM,SHMT2	25
	GO:0015980	energy derivation by oxidation of organic compounds	2.35E-06	0	COX7C,UQCRQ,COX7A2,NDUFA4,COX7B,UQCRB,NDUFB9,COX8A,NDUFB1,NUPR1,COX4I1,UQCR11,COX5A,MRPS36,PID1,CAT,NDUFA1,DNAJC15,NDUFA7,ARL2,NDUFA3,COX6A1,COX6C,COX7A2L,GHITM,SHMT2	26
	GO:1903715	regulation of aerobic respiration	0.000244977	0.013	COX7A2,NUPR1,DNAJC15,ARL2,COX7A2L,SHMT2	6
	GO:0002082	regulation of oxidative phosphorylation	0.000421708	0.019	COX7A2,NUPR1,DNAJC15,COX7A2L,SHMT2	5
Transmembrane/vascular transport	GO:1990542	mitochondrial transmembrane transport	0.000213931	0.013	SLC39A8,TOMM7,SLC25A6,SLC25A5,SLC25A3,SMDT1,MPC1,DNAJC15,SLC25A4,TIMM44	10
	GO:1902600	proton transmembrane transport	0.000160534	0.01	COX17,ATP6V0E1,NDUFA4,COX7B,SLC25A5,COX8A,SLC25A3,CHP1,COX4I1,CYB5A,COX5A,SLC25A4,COX7A2L	13
	GO:0015866	ADP transport	0.000233153	0.013	SLC25A6,SLC25A5,SLC25A4,SLC25A24	4
	GO:0006620	post-translational protein targeting to endoplasmic reticulum membrane	0.00100508	0.033	SEC61G,SEC61B,SEC62,SEC63	4
	GO:0065002	intracellular protein transmembrane transport	0.000816706	0.028	TOMM7,SEC61G,DNAJC15,SEC61B,SEC62,TIMM44,SEC63	7
	GO:0071806	protein transmembrane transport	0.000480568	0.02	TOMM7,EDNRB,SEC61G,DNAJC15,SEC61B,SEC62,TIMM44,SEC63	8
	GO:1990748	cellular detoxification	0.001264141	0.037	SOD3,CD36,MGST1,DUOX1,GPX1,ESD,GPX4,CAT,PRDX5,RDH11	10
	GO:0033194	response to hydroperoxide	0.001264415	0.037	CD36,MGST1,GPX1,CD38	4
	GO:0098754	Detoxification	0.002107261	0.05	SOD3,SLC39A8,CD36,MGST1,DUOX1,GPX1,ESD,GPX4,CAT,PRDX5,RDH11	11
	GO:0097237	cellular response to toxic substance	0.00062111	0.023	SOD3,CD36,MGST1,DUOX1,GPX1,ESD,GPX4,CAT,PRDX5,PTPN13,RDH11	11
	GO:0009636	response to toxic substance	0.001108656	0.035	SOD3,SLC39A8,CD36,MGST1,DUOX1,NUPR1,GPX1,MPST,ESD,GPX4,CAT,PRDX5,PTPN13,RDH11,ATF4,SLC6A14	16
	GO:0120162	positive regulation of cold-induced thermogenesis	0.001492528	0.042	ALPL,ACADL,ACSL1,CD36,FABP5,SCD,GJA1,CAV1,EPAS1	9
	GO:0070252	actin-mediated cell contraction	0.000578161	0.022	EPDR1,KCNJ8,PDE4D,GJA1,CAV1,RANGRF,NEDD4L,LIMCH1,EMP2,GPD1L	10
	GO:0016264	gap junction assembly	0.001264415	0.037	HOPX,GJA1,CAV1,IRX3	4
	GO:1903596	regulation of gap junction assembly	0.002137072	0.05	HOPX,CAV1,IRX3	3
	GO:0003018	vascular process in circulatory system	0.000141139	0.009	MFSD2A,SOD3,CD36,FABP5,KCNJ8,SERPINF2,GPX1,EDNRB,SLC38A1,MKKS,CAV1,TJP2,ATP8A1,SLC22A3,AGER,CD38,ATP2B1,SLC6A20,SLC1A5	19
	GO:0010232	vascular transport	0.000134099	0.009	MFSD2A,CD36,FABP5,KCNJ8,SLC38A1,ATP8A1,SLC22A3,AGER,SLC6A20,SLC1A5	10
	GO:1990845	adaptive thermogenesis	0.001661448	0.043	ALPL,ACADL,ACSL1,CD36,SLC25A5,FABP5,SCD,GJA1,CAV1,EPAS1,SLC25A4,ATF4	12
	GO:0015711	organic anion transport	0.000413093	0.019	MFSD2A,SLC39A8,ACSL1,SLCO4C1,CD36,SLC25A6,SLC25A5,FABP5,MGST1,PLA2G1B,PLA2G4F,MPC1,GJA1,CLN3,SLC38A1,SLC51B,PLIN2,SLC22A3,SLC25A4,SLC6A20,BEST1,SLC6A14,SLC1A5,PLA2G10,SLC25A24	25
	GO:1901264	carbohydrate derivative transport	0.0015667	0.043	MFSD2A,SLC25A6,SLC25A5,NPC2,CLN3,SLC25A4,PLA2G10,SLC25A24	8
	GO:0150104	transport across blood-brain barrier	0.000134099	0.009	MFSD2A,CD36,FABP5,KCNJ8,SLC38A1,ATP8A1,SLC22A3,AGER,SLC6A20,SLC1A5	10
	GO:1902305	regulation of sodium ion transmembrane transport	0.000480568	0.02	CHP1,PCSK9,RANGRF,ATP1B3,NEDD4L,STOM,GPD1L,UTRN	8
	GO:2000649	regulation of sodium ion transmembrane transporter activity	0.001010166	0.033	CHP1,PCSK9,RANGRF,NEDD4L,STOM,GPD1L,UTRN	7
RNA metabolic & processing	GO:0097193	intrinsic apoptotic signaling pathway	0.000695396	0.025	RPL11,RPS7,ZNF385B,RPS3,LRRK2,TPT1,NUPR1,RPL26,GPX1,IL20RA,RPS27L,ERP29,CAV1,TRIB3,PTPN1,ATF4,EIF5A,PTPMT1,DAPK2	19
	GO:0034470	ncRNA processing	0.00039924	0.019	RPL11,RPL5,RPS27,RPS7,RPL14,RPL35A,RPL7,RPS6,RPL35,RPS25,RPS24,RPS17,RPL26,RPL27,RPS21,RPS28,RPS16,RPS8,NUDT16,DDX21,NOP10,NSA2,MPHOSPH6,FRG1,FCF1	25
	GO:1904667	negative regulation of ubiquitin protein ligase activity	1.03E-05	0.001	RPL11,RPL5,RPS7,RPS20,RPL23	5
	GO:0051444	negative regulation of ubiquitin-protein transferase activity	0.000273389	0.014	RPL11,RPL5,RPS7,RPS20,RPL23	5
	GO:0140694	non-membrane-bounded organelle assembly	0.001215351	0.037	RPL11,RPL5,RPS27,TTN,RPSA,CCDC69,SQLE,RPS3,RPL6,RPLP0,AKAP13,RPL23A,RPL38,RPS28,RPS5,NEBL,RPS27L,RPL10,CIRBP,CFL2,CHMP3,EZR	22
	GO:1901798	positive regulation of signal transduction by p53 class mediator	0.000140822	0.009	RPL11,RPS7,RPS20,RPL26,RPL23,EIF5A	6
	GO:2001244	positive regulation of intrinsic apoptotic signaling pathway	0.000313993	0.016	RPL11,RPS7,RPS3,NUPR1,RPL26,IL20RA,CAV1,EIF5A	8
	GO:2001242	regulation of intrinsic apoptotic signaling pathway	8.83E-05	0.006	RPL11,RPS7,RPS3,LRRK2,TPT1,NUPR1,RPL26,GPX1,IL20RA,ERP29,CAV1,PTPN1,EIF5A,PTPMT1,DAPK2	15
	GO:0051438	regulation of ubiquitin-protein transferase activity	0.001119516	0.035	RPL11,RPL5,RPS7,RPS20,RPL23,RPS2,TRIB3	7
	GO:0022618	ribonucleoprotein complex assembly	1.71E-06	0	RPL11,RPL5,RPS27,RPSA,SF3B5,RPL6,RPLP0,RPL23A,RPL38,EIF2S2,RPS28,RPL13A,RPS5,RPS27L,RPL10,SNRPF,EIF3L,SNRPD2,SNRPG,EIF3F,SNRPB2	21
	GO:0071826	ribonucleoprotein complex subunit organization	2.95E-06	0	RPL11,RPL5,RPS27,RPSA,SF3B5,RPL6,RPLP0,RPL23A,RPL38,EIF2S2,RPS28,RPL13A,RPS5,RPS27L,RPL10,SNRPF,EIF3L,SNRPD2,SNRPG,EIF3F,SNRPB2	21
	GO:0000027	ribosomal large subunit assembly	3.53E-06	0	RPL11,RPL5,RPL6,RPLP0,RPL23A,RPL38,RPL10	7
	GO:0042273	ribosomal large subunit biogenesis	4.48E-09	0	RPL11,RPL5,RPL14,RPL35A,RPL7,RPL35,RPL6,RPLP0,RPL26,RPL23A,RPL38,RPL10,RSL24D1,NSA2	14
	GO:0000028	ribosomal small subunit assembly	8.32E-06	0.001	RPS27,RPSA,RPL38,RPS28,RPS5,RPS27L	6
	GO:0042274	ribosomal small subunit biogenesis	2.95E-10	0	RPS27,RPS7,RPSA,RPS6,RPS25,RPS24,RPS17,RPL38,RPS21,RPS28,RPS16,RPS5,RPS8,RPS27L,RPS9,FCF1	16
	GO:0042255	ribosome assembly	6.80E-08	0	RPL11,RPL5,RPS27,RPSA,RPL6,RPLP0,RPL23A,RPL38,RPS28,RPS5,RPS27L,RPL10	12
	GO:0016072	rRNA metabolic process	4.77E-08	0	RPL11,RPL5,RPS27,RPS7,RPL14,RPL35A,RPL7,RPS6,RPL35,RPS25,RPS24,RPS17,RPL26,RPL27,RPS21,RPS28,RPS16,RPS8,NUDT16,DDX21,NOP10,NSA2,MPHOSPH6,FRG1,FCF1	25
	GO:0006364	rRNA processing	1.68E-09	0	RPL11,RPL5,RPS27,RPS7,RPL14,RPL35A,RPL7,RPS6,RPL35,RPS25,RPS24,RPS17,RPL26,RPL27,RPS21,RPS28,RPS16,RPS8,NUDT16,DDX21,NOP10,NSA2,MPHOSPH6,FRG1,FCF1	25
	GO:1903241	U2-type prespliceosome assembly	0.000421708	0.019	SF3B5,SNRPF,SNRPD2,SNRPG,SNRPB2	5
	GO:1904666	regulation of ubiquitin protein ligase activity	0.000748422	0.027	RPL11,RPL5,RPS7,RPS20,RPL23	5
	GO:0022613	ribonucleoprotein complex biogenesis	1.78E-12	0	RPL11,RPL5,RPS27,RPS7,RPSA,RPL14,RPL35A,SF3B5,RPL7,RPS6,RPL35,RPS25,RPS24,RPL6,RPLP0,RPS17,RPL26,RPL23A,RPL27,RPL38,EIF2S2,RPS21,RPS28,RPS16,RPL13A,RPS5,RPS8,RPS27L,NUDT16,RPL10,SNRPF,EIF3L,DDX21,RSL24D1,RPS9,SNRPD2,NOP10,NSA2,SNRPG,EIF3F,SNRPB2,MPHOSPH6,FRG1,FCF1	44
	GO:0002181	cytoplasmic translation	2.22E-68	0	RPL22,RPL11,RPL5,RPS27,RPS7,RPS27A,RPL31,RPL37A,RPL32,RPSA,RPL14,RPL35A,RPL9,RPL34,RPS3A,RPS10,RWDD1,RPS12,RPS4X,RPL36A,RPL39,RPS20,RPL7,RPS6,RPL35,RPLP2,RPL27A,RPS13,RPS3,RPS25,RPS24,RPS26,RPL41,RPL6,RPLP0,RPL21,RPS29,RPL4,RPLP1,RPS17,RPS15A,RPL13,RPL26,RPL23A,RPL23,RPL19,RPL27,RPL38,RPL17,EIF2S2,RPS21,RPL36,RPS28,RPS16,RPL18,RPL13A,RPL28,RPS5,RPS2,RPL3,RPS8,RPL24,RPL18A,YBX1,FAU,RPL10,EIF3L,RPS9,RPL15,RPL7A,EIF3F	71
	GO:0042254	ribosome biogenesis	4.31E-13	0	RPL11,RPL5,RPS27,RPS7,RPSA,RPL14,RPL35A,RPL7,RPS6,RPL35,RPS25,RPS24,RPL6,RPLP0,RPS17,RPL26,RPL23A,RPL27,RPL38,RPS21,RPS28,RPS16,RPS5,RPS8,RPS27L,NUDT16,RPL10,DDX21,RSL24D1,RPS9,NOP10,NSA2,MPHOSPH6,FRG1,FCF1	35

Appendix Table S3. Clinical characteristics

	Discovery set		Validation set plasma		Tissue Validation set
	Health(n=30)	Lung(n=60)	Health(n=30)	Lung(n=30)	Tissue/adjacent(n=25)
Age	29.73±5.99	59.2±10.73	51.57 ±4.52	62.23±9.48	62.72±10.21
Sex					
Female	7	32	10	15	11
Male	23	28	20	15	14
	p-value = 0.0131		p-value = 0.2949		
Stage					
I	-	50	-	26	21
II	-	7	-	3	3
III/IV	-	3	-	1	1
Tumor size	-	2.11±1.05	-	2.67±1.07	2.78±1.09
TumorMarker	-	36 Neg	-	17 Neg	14 Neg
		24Pos		13 Pos	11 Pos

Age: mean±sd; Tumor size: mean±sd.

Appendix Table S4. Clinical information of Discovery Cohort

sample_ID	Age	Sex	Histology	tumor marker	Tumorsize(cm)	TNM	Other lung disease	Smoking	EGFR GeneMutant
L1	74	Male	LUAD	NSE	4.9	IIB	old TB	Yes	WT
L2	49	Female	LUAD	0	1.7	IA2	no	No	−
L3	49	Female	LUAD	0	1.1	IA2	no	No	−
L4	72	Male	LUAD	0	1.2	IA2	no	Yes	−
L5	55	Female	LUAD	NSE	2.2	IA3	no	No	−
L6	41	Female	LUAD	CEA	2	IB	no	No	−
L7	73	Female	LUAD	CEA	2.7	IA3	no	No	−
L8	63	Female	LUAD	0	1.5	IA2	no	No	−
L9	62	Female	LUAD	0	1.2	IA2	no	No	−
L10	49	Male	LUAD	0	1	IA1	no	Yes	−
L11	54	Female	LUAD	0	1.2	IA2	no	No	−
L12	36	Female	LUAD	0	1.1	IA2	no	No	WT
L13	49	Male	LUAD	0	1.4	IA2	no	No	EGFR 18 and 20
L14	59	Female	LUAD	0	1.9	IA2	no	No	EGFR 19DEL
L15	64	Female	LUAD	ProGRP/CEA	1.6	IA2	no	No	−
L16	62	Male	LUAD	Cfra21-1/CEA	2.5	IA3	no	Yes	−
L17	56	Male	LUAD	ProGRP	3.1	IB	no	No	EGFR L858R
L18	44	Female	LUAD	0	1.6	IA2	no	No	−
L19	71	Male	LUAD	NSE/CA125	4.3	IIA	no	Yes	WT
L20	46	Female	LUAD	CA125	1.7	IA2	no	No	−
L21	67	Female	LUAD	0	1.7	IA2	no	No	−
L22	66	Male	LUAD	CYFRA21-1/NSE	4.1	IIA	no	Yes	WT
L23	61	Female	LUAD	CYFRA21-1/CEA	2.4	IB	no	No	−
L24	57	Female	LUAD	0	2.6	IA3	no	No	EGFR 18
L25	73	Male	LUAD	CYFRA21-1/NSE/CA199/CA125/CEA	2.3	IV	no	No	EGFR 19DEL and TP53
L26	70	Female	LUAD	NSE/CA125	4.5	IIA	no	No	−
L27	52	Male	LUAD	CYFRA21-1	1.5	IA2	no	Yes	EFGR 18 and TP53
L28	45	Female	LUAD	0	2.6	IA3	no	No	−
L29	54	Female	LUAD	0	2.2	IA3	no	No	−
L30	69	Female	LUAD	CEA	4.3	IIA	no	No	−
L31	70	Female	LUAD	0	4.1	IIA	no	No	−
L32	49	Female	LUAD	0	1	IA1	no	No	−
L33	79	Female	LUAD	0	2.6	IA3	no	No	WT
L34	57	Female	LUAD	CEA	0.6	IA1	no	No	−
L35	51	Male	LUAD	NSE	1.9	IA2	no	No	EGFR L858R
L36	57	Female	LUAD	CYFRA21-1/NSE	1	IA1	no	No	−
L37	66	Female	LUAD	0	1.9	IA2	no	No	−
L38	54	Male	LUAD	0	2	IA2	no	Yes	−
L39	72	Male	LUAD	0	3.2	IB	no	No	EGFR L858R
L40	70	Male	LUAD	0	0.8	IB	COPD	Yes	−
L41	54	Female	LUAD	NSE	3	IA3	no	No	−
L42	55	Female	LUAD	0	2.1	IIIA	no	No	−
L43	56	Male	LUAD	0	1.7	IA2	no	No	−
L44	57	Male	LUAD	NSE/CEA	2	IV	no	No	EGFR 19DEL and TP53
L45	33	Male	LUAD	0	1.1	IA2	no	No	−
L46	65	Male	LUAD	CYFRA21-1	4.6	IIA	no	No	EFGR 19DEL
L47	80	Male	LUAD	0	2.9	IA3	no	Yes	−
L48	52	Female	LUAD	NSE	2.2	IA3	no	No	−
L49	75	Male	LUAD	CA125	1.7	IA2	no	Yes	−
L50	66	Male	LUAD	0	1.4	IA2	no	Yes	−
L51	49	Male	LUAD	0	2.5	IA3	no	Yes	−
L52	51	Male	LUAD	0	1.2	IA2	no	No	−
L53	69	Female	LUAD	0	2.7	IA3	no	No	−
L54	79	Male	LUAD	0	2.8	IA3	no	Yes	−
L55	59	Female	LUAD	0	1.8	IA2	no	No	−
L56	68	Male	LUAD	0	1.5	IA1	no	Yes	−
L57	51	Female	LUAD	CEA	0.7	IA1	no	No	−
L58	59	Male	LUAD	0	1.3	IA1	no	Yes	−
L59	52	Male	LUAD	0	1.4	IA1	no	No	−
L60	55	Male	LUAD	0	0.9	IA1	COPD	Yes	−
H1	34	Male	Health	−	−	−	−	−	−
H2	34	Male	Health	−	−	−	−	−	−
H3	32	Male	Health	−	−	−	−	−	−
H4	32	Male	Health	−	−	−	−	−	−
H5	21	Female	Health	−	−	−	−	−	−
H6	38	Male	Health	−	−	−	−	−	−
H7	22	Male	Health	−	−	−	−	−	−
H8	29	Male	Health	−	−	−	−	−	−
H9	35	Male	Health	−	−	−	−	−	−
H10	34	Male	Health	−	−	−	−	−	−
H11	30	Male	Health	−	−	−	−	−	−
H12	32	Male	Health	−	−	−	−	−	−
H13	30	Male	Health	−	−	−	−	−	−
H14	21	Male	Health	−	−	−	−	−	−
H15	24	Male	Health	−	−	−	−	−	−
H16	37	Male	Health	−	−	−	−	−	−
H17	35	Male	Health	−	−	−	−	−	−
H18	34	Male	Health	−	−	−	−	−	−
H19	38	Female	Health	−	−	−	−	−	−
H20	37	Female	Health	−	−	−	−	−	−
H21	20	Male	Health	−	−	−	−	−	−
H22	28	Female	Health	−	−	−	−	−	−
H23	22	Female	Health	−	−	−	−	−	−
H24	24	Male	Health	−	−	−	−	−	−
H25	32	Male	Health	−	−	−	−	−	−
H26	18	Male	Health	−	−	−	−	−	−
H27	34	Male	Health	−	−	−	−	−	−
H28	23	Female	Health	−	−	−	−	−	−
H29	33	Male	Health	−	−	−	−	−	−
H30	29	Female	Health	−	−	−	−	−	−

Appendix Table S5. Clinical information of Plasma validation cohort and Tissue validation cohort

Tumor	Adjacent lung tissue	Blood	Age	Sex	Histology	tumor marker	BMI	Tumorsize(cm)	TNM	Other lung conditions	Smoking	EGFR GeneMutant
T1	TN1	L61	64	Male	LUAD	0	24.02381	1.9	IA2	no	Y	WT
T2	TN2	L62	66	Male	LUAD	Cyfra 21-1	22.40588	2.5	IA3	no	Y	WT
T3	TN3	L63	74	Male	LUAD	0	24.09297	3	IIB	no	Y	−
T4	TN4	L64	67	Male	LUAD	0	22.40879	2.7	IA3	COPD	Y	−
T5	−	L65	57	Female	LUAD	0	20.93212	1.9	IB	no	N	−
T7	TN7	L67	50	Male	LUAD	CEA、CA-199	21.45329	2.6	IB	no	N	−
T8	−	L68	58	Female	LUAD	Cyfra 21-1、NSE	20.81165	2.3	IA3	no	N	−
T9	TN9	L69	71	Male	LUAD	Cyfra 21-1、CEA	25.71166	3.2	IB	COPD	N	−
T10	TN10	L70	74	Female	LUAD	ProGRP	19.37716	1.3	IA2	no	N	−
T11	TN11	L71	66	Female	LUAD	Cyfra 21-1	19.84127	2	IA2	no	N	−
T12	TN12	L72	68	Female	LUAD	NSE	27.47138	3.3	IB	no	N	L858R
T13	TN13	L73	60	Female	LUAD	0	21.33333	2	IA2	no	N	−
T14	TN14	L74	72	Female	LUAD	NSE	21.64127	1.9	IA2	bronchiectasis	N	−
T16	TN16	L76	61	Female	LUAD	CA-199	27.09925	3.1	IB	no	N	−
T17	TN17	L77	56	Male	LUAD	0	21.22449	1.9	IA2	no	N	−
T18	TN18	L78	41	Male	LUAD	0	24.38272	1.9	IA2	no	N	L861Q
T19	−	L79	60	Male	LUAD	ProGRP	24.09297	3.2	IB	no	N	−
T20	TN20	L80	61	Female	LUAD	Cyfra 21-1	27.2173	2.3	IA2	no	N	−
T22	TN22	L82	66	Male	LUAD	Cyfra 21-1、ProGRP	24.22145	4.5	IIA	COPD	Y	WT
T23	TN23	L83	46	Male	LUAD	0	25.46939	2.8	IA3	no	Y	−
T24	TN24	L84	59	Female	LUAD	0	22.26563	2.6	IA3	no	N	−
T25	TN25	L85	69	Female	LUAD	0	26.7094	6.4	IIIB	no	N	−
T26	TN26	L86	51	Male	LUAD	0	19.72318	1.5	IA2	no	Y	−
T27	−	L87	67	Female	LUAD	0	18.96193	1.8	IA2	no	N	L858R
T29	TN29	L89	67	Male	LUAD	0	20.76125	3.2	IB	no	N	−
T30	−	L90	57	Female	LUAD	0	24.65483	1.1	IA2	no	N	−
T31	TN31	L91	74	Male	LUAD	0	18.59113	3.6	IB	no	N	WT
T32	TN32	L92	48	Male	LUAD	0	24.56747	2.6	IA3	no	N	−
T33	TN33	L93	54	Female	LUAD	0	22.20633	2.6	IA3	no	N	19DEL
T34	TN34	L94	83	Female	LUAD	Cyfra 21-1、NSE	15.79431	4.4	IIA	no	N	L858R
		N1	47	Male	Health	−	−	−	−			
		N2	53	Male	Health	−	−	−	−			
		N3	47	Male	Health	−	−	−	−			
		N4	57	Male	Health	−	−	−	−			
		N5	47	Female	Health	−	−	−	−			
		N6	57	Female	Health	−	−	−	−			
		N7	50	Female	Health	−	−	−	−			
		N8	51	Male	Health	−	−	−	−			
		N9	47	Male	Health	−	−	−	−			
		N10	48	Male	Health	−	−	−	−			
		N11	58	Female	Health	−	−	−	−			
		N31	50	Female	Health	−	−	−	−			
		N13	54	Female	Health	−	−	−	−			
		N14	47	Male	Health	−	−	−	−			
		N15	50	Male	Health	−	−	−	−			
		N16	48	Male	Health	−	−	−	−			
		N17	61	Male	Health	−	−	−	−			
		N18	47	Female	Health	−	−	−	−			
		N19	59	Male	Health	−	−	−	−			
		N20	51	Male	Health	−	−	−	−			
		N21	58	Female	Health	−	−	−	−			
		N22	50	Female	Health	−	−	−	−			
		N23	54	Female	Health	−	−	−	−			
		N24	47	Male	Health	−	−	−	−			
		N25	50	Male	Health	−	−	−	−			
		N26	48	Male	Health	−	−	−	−			
		N27	48	Male	Health	−	−	−	−			
		N28	57	Male	Health	−	−	−	−			
		N29	48	Male	Health	−	−	−	−			
		N30	58	Male	Health	−	−	−	−			

Appendix Table S6. The level of PE(18:0/18:1) in cell assay.

	Group	LUAD cells		healthy controls		Liver cancer
	Three replicates	A549	NCI-H1975	BEAS-2B	HSF	HepG2
PE 18:0-18:1 level	Rep-1	10446	12561.6	5402.16	7148	6389.61
	Rep-2	8959.13	12602	6312.46	3944.08	7837.88
	Rep-3	10771.2	9352.64	6558.89	4924.27	7268.29
Log2 PE 18:0-18:1 level	Rep-1	13.351	13.617	12.399	12.803	12.642
	Rep-2	13.129	13.621	12.624	11.945	12.936
	Rep-3	13.395	13.191	12.679	12.266	12.827

Appendix Table S7. KEGG pathways involved by PE(18:0.18:1)

Name	KEGGID	Pathway
PE(18:0/18:1)	C00350	map00564 Glycerophospholipid metabolism
		map00563 Glycosylphosphatidylinositol (GPI)-anchor biosynthesis
		* map01100 Metabolic pathways
		* map01110 Biosynthesis of secondary metabolites
		map04136 Autophagy - other
		* map04138 Autophagy - yeast
		map04140 Autophagy - animal
		map04723 Retrograde endocannabinoid signaling
		map05130 Pathogenic Escherichia coli infection
		map05167 Kaposi sarcoma-associated herpesvirus infection

* No related gene lists for pathway

Appendix Table S8. Clinical information of TCGA-LUAD

case_submitter_id	Age	Gender	vital_status	OSday	Stage	PharmaceuticalTherapy	RadiationTherapy	hsa00564	KMgroup
TCGA-62-A471	64	male	Alive	1246	Stage IIB	yes	no	0.5539498	0
TCGA-67-3773	84	female	Alive	427	Stage IB	not reported	not reported	0.5729705	1
TCGA-NJ-A7XG	49	male	Alive	617	Stage IIIA	yes	no	0.5795397	1
TCGA-91-6848	59	male	Alive	224	Stage IIIA	yes	no	0.5311874	0
TCGA-55-6986	74	female	Alive	3261	Stage IB	no	no	0.5625464	0
TCGA-86-6851	73	female	Alive	179	Stage IIA	no	no	0.5585182	0
TCGA-73-4677	74	male	Dead	38	'--	no	no	0.5789872	1
TCGA-78-7540	66	female	Dead	1197	Stage IB	no	no	0.5830372	1
TCGA-55-7576	54	male	Alive	670	Stage IB	yes	no	0.5596889	0
TCGA-55-8615	67	male	Alive	446	Stage IIIA	yes	yes	0.5934563	1
TCGA-49-4501	67	female	Dead	1421	Stage IB	no	no	0.5720109	1
TCGA-86-7954	68	female	Alive	605	Stage IB	yes	no	0.5701514	1
TCGA-44-2655	65	female	Alive	1324	Stage IA	no	no	0.5804617	1
TCGA-49-4505	61	female	Dead	428	Stage IIB	no	no	0.5533396	0
TCGA-97-8177	59	female	Alive	499	Stage IB	yes	no	0.5793113	1
TCGA-J2-A4AD	61	female	Dead	550	Stage IA	no	no	0.5585916	0
TCGA-50-6592	71	female	Dead	777	Stage IB	no	yes	0.5676661	1
TCGA-86-8674	50	male	Alive	806	Stage IIA	yes	no	0.5596747	0
TCGA-55-6981	53	female	Dead	1379	Stage IIIA	yes	yes	0.5711026	1
TCGA-91-6836	52	female	Alive	417	Stage IB	no	no	0.5323815	0
TCGA-62-A46P	65	male	Dead	594	Stage IB	yes	no	0.5929421	1
TCGA-86-7955	62	male	Alive	1072	Stage IB	yes	yes	0.5691475	1
TCGA-50-6597	79	female	Dead	1268	Stage IB	no	no	0.5585893	0
TCGA-91-A4BD	78	male	Alive	603	Stage IIA	no	no	0.5683847	1
TCGA-67-3771	77	female	Alive	610	Stage IA	not reported	not reported	0.5731184	1
TCGA-38-4625	66	female	Alive	2973	Stage IB	no	no	0.5568787	0
TCGA-50-5935	86	female	Dead	653	Stage IA	no	yes	0.5667152	1
TCGA-55-8089	56	male	Dead	702	Stage IA	no	no	0.5631993	1
TCGA-67-6217	73	female	Alive	422	Stage IIA	no	no	0.576975	1
TCGA-50-6590	72	female	Dead	1288	Stage IB	yes	no	0.5316598	0
TCGA-38-4629	68	male	Dead	864	Stage IIB	no	no	0.5483047	0
TCGA-50-5939	85	male	Dead	460	Stage IB	no	no	0.5560156	0
TCGA-55-A48Y	69	male	Alive	630	Stage IIA	yes	no	0.5482278	0
TCGA-50-5931	75	female	Dead	434	Stage IB	no	no	0.5354072	0
TCGA-91-6829	78	male	Dead	1258	Stage IB	no	no	0.5383761	0
TCGA-49-6744	64	female	Alive	1683	Stage IIA	yes	no	0.5658642	1
TCGA-50-5072	74	male	Dead	250	Stage IIIA	yes	yes	0.5515151	0
TCGA-MP-A4SW	53	male	Dead	1778	Stage IIB	no	no	0.585257	1
TCGA-78-7162	75	male	Dead	3169	Stage IA	no	no	0.5752902	1
TCGA-55-A48Z	60	female	Alive	651	Stage IIIB	yes	yes	0.5535946	0
TCGA-78-8662	53	female	Dead	3361	Stage IB	no	yes	0.5638584	1
TCGA-MN-A4N5	63	male	Alive	84	Stage IA	no	no	0.5756331	1
TCGA-78-8640	59	male	Alive	7062	Stage IIA	no	no	0.5662271	1
TCGA-95-7562	71	male	Dead	87	Stage IIA	yes	no	0.5226853	0
TCGA-73-4676	45	male	Dead	281	Stage IIA	yes	yes	0.5664465	1
TCGA-55-1596	55	male	Alive	2065	Stage IIB	yes	no	0.567334	1
TCGA-64-1680	63	male	Alive	1126	Stage IV	yes	yes	0.5740222	1
TCGA-97-7552	70	male	Alive	1932	Stage IB	no	no	0.5727755	1
TCGA-69-7763	69	male	Alive	690	Stage IA	no	no	0.5720138	1
TCGA-73-4670	69	female	Alive	131	Stage IV	yes	no	0.5387992	0
TCGA-75-5146	'--	male	Alive	2368	Stage IB	no	no	0.5756291	1
TCGA-55-7911	70	female	Alive	537	Stage IA	no	no	0.5672093	1
TCGA-55-8511	73	female	Alive	552	Stage IB	no	no	0.5763122	1
TCGA-44-5644	51	female	Alive	863	Stage IB	no	no	0.5527855	0
TCGA-55-8512	41	male	Dead	607	Stage IV	no	no	0.5865045	1
TCGA-75-5125	'--	male	Dead	2027	Stage IIB	no	no	0.5472658	0
TCGA-38-4626	57	female	Alive	3674	'--	no	no	0.5894788	1
TCGA-75-6212	'--	female	Dead	1516	Stage IIB	yes	no	0.5773242	1
TCGA-44-8119	73	male	Alive	285	Stage IIB	no	no	0.5562745	0
TCGA-69-7764	75	male	Alive	414	Stage IA	no	no	0.5822297	1
TCGA-38-4631	72	female	Dead	354	Stage IB	no	no	0.5497645	0
TCGA-83-5908	59	female	Alive	824	Stage IA	no	no	0.5428329	0
TCGA-L4-A4E6	67	male	Alive	435	Stage IA	no	no	0.5935605	1
TCGA-44-5643	53	male	Alive	1013	Stage IIIA	yes	yes	0.5606401	0
TCGA-86-8279	46	male	Alive	949	Stage IIA	yes	yes	0.5634269	1
TCGA-55-7724	76	female	Alive	705	Stage IB	no	no	0.5522397	0
TCGA-73-4662	65	female	Alive	2515	Stage IA	no	no	0.5711841	1
TCGA-73-A9RS	41	male	Dead	340	Stage IIB	yes	yes	0.5407713	0
TCGA-L9-A5IP	40	female	Dead	58	Stage IV	no	no	0.54539	0
TCGA-J2-8194	69	female	Alive	724	Stage IIB	yes	yes	0.567334	1
TCGA-55-8299	61	female	Dead	469	Stage IA	no	yes	0.5510354	0
TCGA-75-6211	'--	female	Dead	'--	Stage IB	no	no	'--	'--
TCGA-78-7149	71	male	Alive	3940	Stage IIIB	no	no	0.5715975	1
TCGA-55-6987	77	male	Alive	2137	Stage IA	no	no	0.5573168	0
TCGA-55-6980	56	male	Alive	2109	Stage IA	no	no	0.5584169	0
TCGA-91-6835	81	female	Alive	79	Stage IA	no	no	0.5597899	0
TCGA-86-8585	57	male	Alive	353	Stage IB	no	no	0.5555254	0
TCGA-62-8398	55	male	Dead	444	Stage IIIA	yes	no	0.5609406	0
TCGA-MP-A4T4	68	female	Dead	2617	Stage IIB	no	no	0.5573668	0
TCGA-93-A4JP	64	male	Alive	578	Stage IV	yes	yes	0.5633469	1
TCGA-S2-AA1A	68	female	Alive	513	Stage IA	no	no	0.5772934	1
TCGA-50-5066	72	male	Alive	1442	Stage IB	yes	no	0.543926	0
TCGA-05-4398	47	female	Alive	1431	Stage IIIB	yes	no	0.5573852	0
TCGA-97-A4M6	45	female	Alive	568	Stage IA	no	no	0.5773626	1
TCGA-75-6214	'--	female	Dead	1115	Stage IIIA	no	yes	0.56095	0
TCGA-L4-A4E5	48	female	Alive	578	Stage I	no	no	0.5555694	0
TCGA-55-A48X	63	female	Alive	689	Stage IIA	yes	no	0.5765043	1
TCGA-44-6774	56	female	Alive	658	Stage IIIA	yes	yes	0.5523255	0
TCGA-05-5423	65	male	Alive	151	Stage IIB	not reported	not reported	0.5677524	1
TCGA-05-4244	70	male	Alive	0	Stage IV	not reported	not reported	0.5781341	1
TCGA-05-4382	68	male	Alive	607	Stage IB	no	yes	0.5584835	0
TCGA-97-A4M2	66	male	Alive	624	Stage IA	no	no	0.5990049	1
TCGA-44-6777	85	female	Dead	987	Stage IB	no	yes	0.5556109	0
TCGA-86-8055	79	male	Dead	124	Stage IIA	no	no	0.5454221	0
TCGA-55-6982	79	female	Dead	995	Stage IIB	yes	yes	0.5680461	1
TCGA-55-A4DF	88	male	Dead	440	Stage IA	no	yes	0.5545024	0
TCGA-55-8096	67	female	Dead	719	Stage IB	no	yes	0.5675077	1
TCGA-50-5930	47	male	Dead	282	Stage IIIA	yes	yes	0.552195	0
TCGA-J2-8192	65	female	Alive	739	Stage IIA	yes	no	0.5551134	0

case_submitter_id	Age	Gender	vital_status	OSday	Stage	PharmaceuticalTherapy	RadiationTherapy	hsa00564	KMgroup
TCGA-67-3774	73	female	Alive	385	Stage IB	not reported	not reported	0.5846691	1
TCGA-05-4426	71	male	Alive	791	Stage IB	not reported	not reported	0.5462254	0
TCGA-71-8520	60	female	Dead	210	Stage IB	no	yes	0.5870307	1
TCGA-97-7553	58	female	Alive	1870	Stage IA	no	no	0.5698024	1
TCGA-MP-A4TD	71	male	Dead	307	Stage IIIA	yes	no	0.5761909	1
TCGA-95-A4VN	62	female	Alive	553	Stage IIA	no	no	0.5564216	0
TCGA-97-8171	81	male	Alive	568	Stage IV	yes	no	0.5676474	1
TCGA-55-A492	70	female	Alive	596	Stage IA	no	no	0.5957205	1
TCGA-05-4430	59	female	Alive	761	Stage IB	not reported	not reported	0.5651522	1
TCGA-49-AAR0	57	male	Alive	4765	Stage IA	no	no	0.575721	1
TCGA-55-A57B	80	female	Alive	546	Stage IA	no	no	0.5602486	0
TCGA-L9-A443	63	female	Dead	193	Stage IA	no	no	0.5727589	1
TCGA-95-7567	61	male	Alive	568	Stage IIB	yes	no	0.5598752	0
TCGA-44-3396	74	female	Alive	1130	Stage IIIA	yes	no	0.5462257	0
TCGA-44-6775	72	female	Alive	705	Stage IB	no	no	0.5555523	0
TCGA-05-4420	41	male	Alive	912	Stage IB	no	no	0.5562443	0
TCGA-93-A4JO	70	male	Dead	33	Stage IA	no	no	0.5803533	1
TCGA-69-7974	54	female	Alive	184	Stage IIIA	yes	yes	0.5630801	1
TCGA-55-A4DG	71	male	Alive	608	Stage IA	no	no	0.579515	1
TCGA-55-8506	62	female	Alive	11	Stage IIB	not reported	not reported	0.5651177	1
TCGA-05-5428	57	male	Alive	670	Stage IIA	yes	yes	0.5603122	0
TCGA-44-7672	52	female	Alive	719	Stage IA	no	no	0.5609285	0
TCGA-64-5781	55	female	Alive	1559	Stage IB	no	no	0.5330057	0
TCGA-NJ-A4YP	52	male	Alive	50	Stage IB	no	no	0.5609182	0
TCGA-O1-A52J	74	female	Dead	1798	Stage IA	no	no	0.5772487	1
TCGA-44-2656	59	male	Alive	1429	Stage IB	no	no	0.5478679	0
TCGA-53-7813	51	female	Alive	424	Stage IIIB	yes	no	0.5633787	1
TCGA-62-8402	73	female	Dead	1498	Stage IIIA	yes	yes	0.5636363	1
TCGA-78-7633	67	male	Dead	1528	Stage IB	not reported	not reported	0.5937778	1
TCGA-35-4122	69	male	Alive	225	Stage IA	not reported	not reported	0.5560761	0
TCGA-69-8253	59	female	Alive	426	Stage IIA	yes	no	0.5867984	1
TCGA-05-4422	68	male	Alive	365	Stage IB	not reported	not reported	0.5891631	1
TCGA-78-7535	45	male	Dead	949	Stage IB	yes	no	0.5710341	1
TCGA-86-8358	44	male	Alive	653	Stage IB	no	no	0.532096	0
TCGA-MP-A4T7	75	female	Dead	167	Stage IV	no	no	0.5773976	1
TCGA-86-8056	63	female	Alive	139	Stage IIIA	not reported	not reported	0.5827238	1
TCGA-55-6642	63	male	Alive	2449	Stage IB	no	no	0.5500018	0
TCGA-49-AAR9	61	male	Dead	260	Stage IIB	no	no	0.5550215	0
TCGA-86-8074	62	female	Alive	24	Stage IIA	not reported	not reported	0.5336814	0
TCGA-75-7027	'--	male	Alive	3059	Stage IB	yes	yes	0.5479366	0
TCGA-50-8460	74	male	Alive	829	Stage IA	no	yes	0.5768729	1
TCGA-L9-A444	60	female	Alive	307	Stage IA	no	no	0.5606214	0
TCGA-91-8497	75	female	Dead	434	Stage IA	no	no	0.5849123	1
TCGA-05-4249	67	male	Alive	1523	Stage IB	no	no	0.5705752	1
TCGA-44-7669	59	male	Dead	574	Stage IIA	yes	no	0.5582596	0
TCGA-71-6725	48	female	Alive	256	Stage IB	yes	yes	0.5548681	0
TCGA-55-8092	75	male	Dead	154	Stage IIB	no	no	0.5354738	0
TCGA-73-4666	52	female	Alive	800	Stage IV	yes	yes	0.5662297	1
TCGA-49-6761	68	female	Alive	354	Stage IIIA	no	no	0.5442765	0
TCGA-78-7159	60	female	Alive	1974	Stage IA	no	no	0.5656774	1
TCGA-55-8090	80	male	Dead	598	Stage IA	no	no	0.5595773	0
TCGA-55-7227	77	male	Dead	952	Stage IIIA	yes	no	0.5508144	0
TCGA-78-7145	52	female	Dead	826	Stage IV	yes	no	0.5536735	0
TCGA-75-7031	'--	female	Alive	'--	Stage IB	no	no	'--	'--
TCGA-78-7158	59	female	Dead	179	Stage IIIB	yes	no	0.554254	0
TCGA-49-4507	73	female	Dead	268	Stage IIIA	yes	yes	0.5366497	0
TCGA-55-6978	81	male	Dead	176	Stage IIA	no	no	0.5429913	0
TCGA-49-AARO	39	female	Alive	3759	Stage IA	yes	yes	0.5636018	1
TCGA-86-A4D0	48	male	Dead	116	Stage IIA	yes	no	0.5741235	1
TCGA-NJ-A4YQ	69	female	Alive	1432	Stage IA	no	no	0.5553844	0
TCGA-86-7714	61	female	Dead	625	Stage IIIA	no	no	0.5773416	1
TCGA-78-7143	62	female	Dead	4961	Stage IB	no	no	0.5554409	0
TCGA-91-8499	76	female	Alive	36	Stage IA	no	no	0.5366881	0
TCGA-86-7953	69	female	Alive	997	Stage IA	no	no	0.5633956	1
TCGA-55-7816	49	female	Dead	468	Stage IV	no	no	0.55467	0
TCGA-53-7624	40	female	Dead	1043	Stage IV	yes	yes	0.5441762	0
TCGA-L9-A7SV	69	male	Alive	565	Stage IIA	yes	no	0.560048	0
TCGA-55-8620	60	male	Dead	375	Stage IV	no	yes	0.555247	0
TCGA-69-8255	71	male	Alive	129	Stage IA	no	no	0.561961	0
TCGA-62-A46Y	70	female	Dead	414	Stage IIIA	yes	yes	0.5676927	1
TCGA-97-A4M3	69	female	Alive	540	Stage IA	no	no	0.562303	0
TCGA-49-AAR3	69	male	Alive	1893	Stage IIB	yes	no	0.5497995	0
TCGA-75-6207	'--	male	Dead	'--	Stage IIIA	no	no	'--	'--
TCGA-50-5068	59	female	Dead	1499	Stage IIB	yes	no	0.551829	0
TCGA-NJ-A4YF	50	female	Alive	2161	Stage IA	no	no	0.5557899	0
TCGA-91-6830	65	female	Alive	60	Stage IIA	no	yes	0.5594942	0
TCGA-78-7163	60	male	Alive	7248	Stage IB	no	no	0.5619455	0
TCGA-78-8648	58	female	Dead	1209	Stage IIB	no	no	0.5717043	1
TCGA-55-6979	59	female	Dead	237	Stage IIB	yes	yes	0.553483	0
TCGA-80-5608	'--	female	Alive	2832	Stage IA	no	no	0.5838448	1
TCGA-62-8395	80	female	Alive	1216	Stage IIB	yes	no	0.6004697	1
TCGA-35-5375	61	male	Alive	264	Stage IIIA	not reported	not reported	0.5352867	0
TCGA-44-6778	59	male	Alive	1864	Stage IA	no	no	0.5558941	0
TCGA-86-8281	75	male	Alive	0	Stage IA	not reported	not reported	0.5840453	1
TCGA-MP-A4T6	76	female	Dead	1790	Stage IIIA	no	no	0.5882674	1
TCGA-55-8085	64	male	Alive	904	Stage IA	no	no	0.5682287	1
TCGA-97-A4M5	83	male	Alive	634	Stage IA	no	no	0.5842195	1
TCGA-97-7547	67	female	Alive	1965	Stage IB	yes	no	0.5970638	1
TCGA-44-6145	62	female	Alive	595	Stage IA	no	no	0.5508967	0
TCGA-49-4486	72	male	Dead	2318	Stage IA	no	no	0.5753786	1
TCGA-62-8399	62	male	Alive	2696	Stage IIIA	no	no	0.5491604	0
TCGA-05-5429	60	male	Dead	275	Stage IIIA	not reported	not reported	0.5617931	0
TCGA-80-5607	'--	female	Alive	'--	Stage IIB	no	no	'--	'--
TCGA-86-A4P8	59	female	Alive	805	Stage IIIA	yes	yes	0.5875122	1
TCGA-91-8496	63	female	Alive	505	Stage IB	no	no	0.5761433	1
TCGA-55-7281	70	female	Alive	872	Stage IA	yes	yes	0.5754284	1
TCGA-05-4424	70	male	Alive	913	Stage IIB	yes	yes	0.5688812	1
TCGA-44-3919	71	female	Dead	1026	Stage IA	no	yes	0.5679082	1
TCGA-67-3772	82	female	Alive	573	Stage IB	not reported	not reported	0.5691375	1
TCGA-78-7537	72	male	Dead	1622	Stage IB	no	no	0.5635821	1
TCGA-55-7994	81	male	Alive	603	Stage IIB	yes	no	0.5667933	1

case_submitter_id	Age	Gender	vital_status	OSday	Stage	PharmaceuticalTherapy	RadiationTherapy	hsa00564	KMgroup
TCGA-69-A59K	60	female	Alive	591	Stage IIB	yes	no	0.5625854	1
TCGA-75-7025	'--	male	Alive	3305	Stage IB	yes	no	0.5750468	1
TCGA-55-8514	70	female	Alive	520	Stage IB	no	no	0.5928011	1
TCGA-44-4112	60	female	Dead	808	Stage IB	yes	no	0.5615566	0
TCGA-55-8094	51	male	Alive	541	Stage IV	yes	yes	0.5645878	1
TCGA-05-4425	70	female	Alive	669	Stage IV	not reported	not reported	0.5667152	1
TCGA-55-7914	71	female	Dead	187	Stage IIA	yes	no	0.5691238	1
TCGA-49-6767	46	female	Alive	677	Stage IIB	not reported	not reported	0.5480647	0
TCGA-49-4490	45	female	Dead	385	Stage IIIA	yes	yes	0.5604091	0
TCGA-35-4123	38	male	Alive	182	Stage IA	not reported	not reported	0.5452563	0
TCGA-55-1594	68	male	Alive	1178	Stage IIIA	not reported	not reported	0.5712099	1
TCGA-44-8117	54	female	Alive	385	Stage IB	yes	no	0.5680229	1
TCGA-05-4397	65	male	Dead	731	Stage IIB	not reported	not reported	0.5641015	1
TCGA-44-3918	60	female	Alive	1036	Stage IA	yes	yes	0.5530162	0
TCGA-95-8494	67	male	Alive	84	Stage IIA	no	no	0.5476366	0
TCGA-MP-A4T9	54	female	Dead	1265	Stage IIIA	yes	yes	0.5706034	1
TCGA-MP-A4SV	67	male	Dead	2620	Stage IB	no	no	0.561329	0
TCGA-44-3917	33	female	Alive	1183	Stage IB	no	no	0.5414284	0
TCGA-05-4402	57	female	Dead	244	Stage IV	yes	no	0.5634771	1
TCGA-62-8394	65	female	Dead	139	Stage IIIB	yes	no	0.5527199	0
TCGA-55-8204	87	female	Alive	515	Stage IB	no	no	0.5353559	0
TCGA-MN-A4N1	60	male	Alive	827	Stage IIA	no	no	0.5619573	0
TCGA-50-5942	67	female	Alive	1847	Stage IA	no	yes	0.5614284	0
TCGA-05-4395	76	male	Dead	0	Stage IIIB	not reported	not reported	0.5561595	0
TCGA-49-AAR4	51	male	Dead	879	Stage IIIA	yes	yes	0.5619147	0
TCGA-73-7499	81	female	Dead	1531	Stage IB	no	no	0.5340747	0
TCGA-55-7903	64	male	Alive	567	Stage IA	no	no	0.5595628	0
TCGA-55-8208	73	female	Alive	674	Stage IA	no	yes	0.5610337	0
TCGA-78-8660	69	male	Dead	321	Stage IIB	no	yes	0.5719898	1
TCGA-05-4427	65	female	Alive	791	Stage IIB	yes	no	0.5463228	0
TCGA-75-5122	'--	male	Dead	'--	Stage IB	no	no	'--	'--
TCGA-62-A46U	71	female	Alive	2067	Stage IIB	yes	no	0.5777115	1
TCGA-50-5933	72	male	Dead	2393	Stage IIIB	yes	yes	0.5378927	0
TCGA-MP-A4TH	70	female	Alive	741	Stage IA	no	no	0.577921	1
TCGA-78-7156	62	male	Dead	976	Stage IV	no	no	0.5711278	1
TCGA-86-8669	64	male	Alive	938	Stage IA	yes	no	0.5951469	1
TCGA-44-A47A	78	female	Alive	466	Stage IB	no	yes	0.5866102	1
TCGA-78-7539	75	female	Alive	791	Stage IIA	yes	yes	0.5635187	1
TCGA-MP-A4T8	68	male	Dead	161	Stage IIIA	yes	no	0.5470138	0
TCGA-NJ-A55R	67	male	Alive	603	Stage IA	no	no	0.5666554	1
TCGA-55-6970	67	female	Dead	464	Stage IIIA	yes	yes	0.5612882	0
TCGA-86-8359	52	male	Dead	444	Stage IIIA	not reported	not reported	0.5615992	0
TCGA-99-8032	61	male	Alive	44	Stage IA	no	no	0.5664934	1
TCGA-50-7109	60	male	Dead	308	Stage IA	no	no	0.5472995	0
TCGA-44-7667	49	female	Alive	1097	Stage IIB	no	no	0.5352104	0
TCGA-NJ-A4YG	65	male	Alive	2261	Stage IB	no	no	0.5777023	1
TCGA-55-8302	54	male	Alive	478	Stage IB	no	no	0.5634584	1
TCGA-64-1679	58	female	Alive	2488	Stage IIIA	yes	no	0.5394218	0
TCGA-38-7271	72	female	Dead	800	Stage IA	no	no	0.5660579	1
TCGA-50-6595	74	female	Dead	189	Stage IIIA	yes	yes	0.5345426	0
TCGA-55-6968	61	male	Dead	1293	Stage IV	yes	yes	0.5425688	0
TCGA-44-8120	58	male	Alive	260	Stage IB	no	no	0.5627772	1
TCGA-99-8025	72	female	Alive	1060	Stage IIIA	yes	no	0.5475921	0
TCGA-50-6594	79	female	Dead	370	Stage IIIA	no	yes	0.5446648	0
TCGA-44-A479	73	female	Alive	486	Stage IB	no	no	0.5674627	1
TCGA-86-8076	42	male	Alive	993	Stage IA	yes	no	0.5612287	0
TCGA-MP-A4TF	58	female	Dead	336	Stage IIA	yes	yes	0.5371286	0
TCGA-78-7161	69	female	Dead	291	Stage IIB	yes	no	0.5666525	1
TCGA-38-4632	42	male	Dead	1357	Stage IV	no	no	0.5616702	0
TCGA-86-8075	66	female	Dead	694	Stage IB	yes	no	0.5527323	0
TCGA-44-6147	67	female	Alive	845	Stage IA	no	no	0.5435242	0
TCGA-69-7760	73	male	Alive	202	Stage IIB	yes	no	0.5563303	0
TCGA-64-1677	77	female	Dead	628	Stage IIIA	yes	no	0.5473187	0
TCGA-05-5420	67	male	Alive	457	Stage IIIA	not reported	not reported	0.5621765	0
TCGA-97-8174	67	male	Dead	164	Stage IIA	yes	no	0.5900814	1
TCGA-MP-A4SY	61	male	Dead	1501	Stage IIB	yes	no	0.5724482	1
TCGA-55-6971	59	female	Alive	1400	Stage IB	no	no	0.5605367	0
TCGA-55-8621	75	female	Alive	515	Stage IA	no	no	0.5777086	1
TCGA-86-A456	78	female	Alive	896	Stage IA	not reported	no	0.5894691	1
TCGA-55-7573	72	female	Alive	487	Stage IA	no	no	0.5697385	1
TCGA-MP-A5C7	76	female	Alive	2248	Stage IB	no	no	0.5859466	1
TCGA-64-5778	60	male	Alive	1305	Stage IB	no	yes	0.554329	0
TCGA-55-7726	72	female	Alive	652	Stage IA	no	no	0.5177279	0
TCGA-62-8397	70	female	Alive	1289	Stage IIB	no	no	0.564607	1
TCGA-L9-A8F4	64	female	Alive	476	Stage IB	no	yes	0.5557493	0
TCGA-75-5126	'--	female	Alive	'--	Stage IIIA	no	no	'--	'--
TCGA-91-6828	70	male	Alive	323	Stage IA	no	no	0.5561514	0
TCGA-91-6849	75	female	Alive	35	Stage IIIA	not reported	not reported	0.5798715	1
TCGA-99-8033	74	female	Dead	656	Stage IV	yes	yes	0.5658734	1
TCGA-55-8205	76	female	Alive	599	Stage IIA	yes	yes	0.5602857	0
TCGA-55-8510	55	female	Alive	539	Stage IB	no	no	0.5743172	1
TCGA-44-A47B	79	male	Alive	287	Stage IB	no	no	0.5881411	1
TCGA-99-7458	74	female	Alive	747	Stage IIIA	yes	no	0.5616337	0
TCGA-49-AAQV	63	female	Dead	677	Stage II	no	yes	0.5599425	0
TCGA-99-8028	50	female	Alive	1118	Stage IA	no	no	0.5672498	1
TCGA-91-6847	62	female	Alive	842	Stage IB	no	yes	0.5304596	0
TCGA-MP-A4TA	75	female	Dead	950	Stage IA	yes	yes	0.5627204	1
TCGA-49-AARN	56	female	Dead	1135	Stage IA	no	no	0.5666036	1
TCGA-50-5049	70	male	Alive	3094	Stage IA	no	yes	0.5458181	0
TCGA-55-5899	58	male	Alive	930	'--	yes	no	0.5519579	0
TCGA-MP-A4TC	77	male	Dead	74	Stage IIIA	yes	no	0.5479131	0
TCGA-49-AARQ	41	female	Alive	6732	Stage I	no	no	0.5762801	1
TCGA-05-4384	66	male	Alive	426	Stage IIIA	yes	yes	0.5869455	1
TCGA-75-6205	'--	female	Dead	'--	Stage IB	no	no	'--	'--
TCGA-55-8203	69	female	Alive	547	Stage IA	no	no	0.5633695	1
TCGA-50-6591	63	female	Dead	119	Stage IV	no	no	0.5060361	0
TCGA-44-7670	47	female	Alive	882	Stage IIA	yes	no	0.5586498	0
TCGA-05-5425	68	male	Alive	882	Stage IIB	yes	yes	0.5702056	1
TCGA-49-AARR	68	male	Alive	4992	Stage IA	no	no	0.5750182	1
TCGA-49-6745	82	male	Alive	522	Stage IIIA	yes	no	0.5547402	0
TCGA-86-7711	70	male	Dead	1046	Stage IIA	yes	no	0.5637455	1

case_submitter_id	Age	Gender	vital_status	OSday	Stage	PharmaceuticalTherapy	RadiationTherapy	hsa00564	KMgroup
TCGA-97-8552	55	female	Alive	626	Stage I	no	no	0.5837908	1
TCGA-86-A4P7	63	female	Alive	415	Stage IB	no	no	0.5831488	1
TCGA-50-5045	57	female	Dead	2174	'--	yes	yes	0.5575439	0
TCGA-05-4396	76	male	Dead	303	Stage IIIB	not reported	not reported	0.5840455	1
TCGA-55-7725	68	female	Alive	442	Stage IA	no	no	0.5638213	1
TCGA-55-6975	61	male	Dead	118	Stage IIB	no	no	0.5237357	0
TCGA-78-7150	59	male	Dead	666	Stage IIB	no	no	0.5594394	0
TCGA-49-4512	69	female	Dead	905	Stage IIIA	yes	no	0.5563577	0
TCGA-55-8206	56	male	Alive	888	Stage IA	no	no	0.5721393	1
TCGA-05-4417	51	female	Alive	455	Stage IB	no	no	0.5745479	1
TCGA-55-A491	81	female	Alive	626	Stage IA	no	no	0.559771	0
TCGA-49-6743	81	female	Alive	1621	Stage IIIA	yes	no	0.5415833	0
TCGA-05-4389	70	male	Alive	1369	Stage IA	no	no	0.5885305	1
TCGA-86-7701	66	male	Alive	947	Stage IV	yes	yes	0.5615813	0
TCGA-55-1592	65	male	Dead	701	Stage IA	no	no	0.5754213	1
TCGA-95-7948	42	female	Alive	476	Stage IB	no	no	0.5473032	0
TCGA-44-7660	72	male	Alive	592	Stage IB	yes	no	0.5349544	0
TCGA-L9-A743	56	male	Alive	664	Stage IIA	yes	no	0.5727476	1
TCGA-64-5815	74	male	Alive	866	Stage IIB	yes	no	0.5599178	0
TCGA-55-7574	64	female	Dead	995	Stage IB	yes	yes	0.5801491	1
TCGA-L9-A50W	75	male	Dead	442	Stage IIA	no	no	0.5889686	1
TCGA-75-6203	'--	female	Alive	'--	Stage IIIA	yes	no	'--	'--
TCGA-55-8614	76	male	Alive	536	Stage IB	no	no	0.5606235	0
TCGA-78-8655	77	female	Alive	2360	Stage IA	no	no	0.5618889	0
TCGA-97-7554	83	female	Alive	775	Stage IIIA	yes	no	0.5523508	0
TCGA-50-6673	84	female	Dead	22	Stage I	no	yes	0.5646325	1
TCGA-44-7662	61	male	Alive	218	Stage IB	no	no	0.5423219	0
TCGA-95-7944	71	male	Alive	377	Stage IA	no	no	0.5305386	0
TCGA-55-7995	73	female	Alive	889	Stage IA	yes	yes	0.5701871	1
TCGA-73-7498	58	female	Alive	1189	Stage IA	yes	no	0.5875688	1
TCGA-55-A494	61	female	Alive	481	Stage IB	no	no	0.5878048	1
TCGA-73-4675	59	male	Dead	922	Stage IIIA	yes	no	0.5710249	1
TCGA-95-7039	54	female	Alive	1272	Stage IIB	no	no	0.552812	0
TCGA-05-4433	82	male	Alive	730	Stage IB	not reported	not reported	0.5931968	1
TCGA-69-7980	70	female	Alive	411	Stage I	no	no	0.55651	0
TCGA-73-4658	80	female	Dead	1600	Stage IB	no	no	0.565024	1
TCGA-55-8097	60	female	Alive	476	Stage IA	no	no	0.5655869	1
TCGA-73-4668	66	female	Alive	467	Stage IIB	yes	no	0.5657495	1
TCGA-55-7910	50	female	Alive	1040	Stage IIA	yes	no	0.5650446	1
TCGA-55-8301	58	male	Alive	534	Stage IB	yes	yes	0.5651025	1
TCGA-78-7166	84	male	Dead	258	Stage IIB	no	yes	0.5621249	0
TCGA-78-7152	65	male	Dead	1215	Stage IB	no	yes	0.5631211	1
TCGA-49-4506	68	female	Dead	999	Stage IIB	yes	yes	0.5446982	0
TCGA-05-4390	58	female	Alive	1126	Stage IB	yes	no	0.5720832	1
TCGA-44-7659	70	male	Alive	691	Stage IA	no	no	0.5719435	1
TCGA-49-4514	79	female	Alive	1700	Stage IA	no	no	0.5765993	1
TCGA-55-A490	78	male	Dead	99	Stage IIA	yes	no	0.5509759	0
TCGA-55-7727	70	male	Alive	119	Stage IIIA	yes	no	0.5604559	0
TCGA-44-2668	51	male	Dead	761	Stage IB	no	no	0.5341608	0
TCGA-93-8067	77	male	Alive	186	Stage IB	no	no	0.5598668	0
TCGA-86-8673	61	male	Alive	862	Stage IB	no	no	0.5391502	0
TCGA-69-7765	56	male	Alive	165	'--	yes	no	0.5652853	1
TCGA-49-4487	72	female	Dead	855	Stage IA	no	yes	0.5484757	0
TCGA-MP-A4TE	56	male	Dead	896	Stage IIA	yes	no	0.5832906	1
TCGA-44-2659	65	female	Alive	1367	Stage IIB	yes	no	0.5562893	0
TCGA-44-A4SU	67	female	Dead	409	Stage IA	no	no	0.5596226	0
TCGA-55-7907	77	male	Dead	343	Stage IIA	no	no	0.5782744	1
TCGA-97-A4M1	52	female	Alive	601	Stage IA	no	no	0.5758194	1
TCGA-86-8672	59	male	Dead	19	Stage IIB	no	no	0.5636232	1
TCGA-91-7771	62	male	Alive	492	Stage IIB	yes	no	0.5693362	1
TCGA-78-7160	61	male	Dead	697	Stage IV	yes	no	0.5649938	1
TCGA-44-6776	60	female	Alive	2616	Stage IA	no	no	0.5961426	1
TCGA-78-7153	65	female	Alive	3635	Stage IB	no	no	0.5772863	1
TCGA-62-A46R	54	female	Dead	1725	Stage IB	no	no	0.5662426	1
TCGA-75-6206	'--	male	Alive	2590	Stage IB	no	no	0.5840892	1
TCGA-86-8671	72	female	Alive	839	Stage IIB	yes	no	0.5824162	1
TCGA-55-6972	72	male	Dead	1632	Stage IB	no	no	0.5648785	1
TCGA-64-1681	61	female	Alive	1167	Stage IA	no	no	0.569568	1
TCGA-95-7043	63	female	Dead	503	Stage IA	yes	no	0.5569976	0
TCGA-97-A4M7	74	male	Alive	629	Stage IA	no	no	0.5758725	1
TCGA-49-4494	77	male	Dead	1081	Stage IIIA	not reported	not reported	0.5560667	0
TCGA-64-5775	71	male	Dead	62	Stage IIIA	no	no	0.5305388	0
TCGA-50-5946	62	male	Alive	1617	Stage IA	yes	yes	0.5593684	0
TCGA-44-6779	50	female	Dead	500	Stage IIB	yes	no	0.541522	0
TCGA-44-7661	69	female	Dead	557	Stage IB	yes	yes	0.5530873	0
TCGA-50-5055	79	female	Dead	1830	Stage IIA	yes	yes	0.5576899	0
TCGA-38-4628	65	female	Dead	1492	Stage IIB	no	no	0.5621328	0
TCGA-86-7713	70	male	Alive	1157	Stage IIA	yes	no	0.5655648	1
TCGA-44-2662	65	male	Alive	1280	Stage IB	yes	no	0.5585369	0
TCGA-67-6216	57	female	Alive	141	Stage IA	no	no	0.5538788	0
TCGA-55-7283	76	female	Alive	609	Stage IIIA	yes	no	0.5612982	0
TCGA-73-4659	66	male	Dead	711	Stage IIIA	yes	yes	0.5795192	1
TCGA-86-8073	58	male	Alive	740	Stage IB	no	no	0.5702129	1
TCGA-49-AARE	51	female	Dead	1229	Stage IA	yes	yes	0.5565558	0
TCGA-05-4403	76	male	Alive	578	Stage IB	not reported	not reported	0.5784617	1
TCGA-MN-A4N4	57	male	Alive	1175	Stage IA	no	no	0.543125	0
TCGA-55-8091	74	male	Alive	600	Stage IB	no	no	0.5560356	0
TCGA-05-4415	57	male	Dead	91	Stage IIIB	no	no	0.5347841	0
TCGA-75-5147	'--	female	Alive	1333	Stage IB	no	no	0.5461168	0
TCGA-78-7154	72	male	Dead	593	Stage IIIA	not reported	not reported	0.5634271	1
TCGA-05-4410	62	male	Alive	0	Stage IB	not reported	not reported	0.5598262	0
TCGA-05-4418	69	male	Dead	274	Stage IIIA	not reported	not reported	0.5432463	0
TCGA-55-1595	74	female	Alive	1479	Stage IA	no	no	0.568611	1
TCGA-95-A4VK	74	female	Alive	651	Stage IIIA	yes	yes	0.5816079	1
TCGA-99-AA5R	70	female	Alive	658	Stage IA	no	no	0.5796476	1
TCGA-53-7626	76	female	Dead	929	Stage IIA	yes	no	0.5617415	0
TCGA-50-8457	63	female	Alive	1125	Stage IA	no	no	0.5656003	1
TCGA-91-6840	59	female	Alive	372	Stage IA	no	no	0.5466128	0
TCGA-55-8507	53	male	Alive	418	Stage IA	no	no	0.5800305	1
TCGA-55-6969	52	male	Alive	1239	Stage IB	not reported	not reported	0.5471656	0
TCGA-38-4627	64	female	Dead	1147	Stage IIA	no	no	0.5504328	0

case_submitter_id	Age	Gender	vital_status	OSday	Stage	PharmaceuticalTherapy	RadiationTherapy	hsa00564	KMgroup
TCGA-49-4488	74	female	Dead	869	Stage IA	no	no	0.5517906	0
TCGA-62-A46O	65	female	Dead	1454	Stage IB	no	no	0.5374809	0
TCGA-86-8280	54	female	Alive	701	Stage IIA	yes	no	0.5676806	1
TCGA-05-4434	67	female	Dead	457	Stage IV	not reported	not reported	0.5665491	1
TCGA-44-A47G	73	female	Alive	351	Stage IA	no	no	0.5754849	1
TCGA-78-7536	69	male	Dead	244	Stage IIIA	no	yes	0.5542382	0
TCGA-91-6831	66	male	Alive	310	Stage IB	no	no	0.5435807	0
TCGA-67-6215	52	female	Alive	174	Stage IB	yes	no	0.5711583	1
TCGA-69-7973	42	female	Alive	230	Stage IB	yes	no	0.5646517	1
TCGA-86-6562	52	male	Dead	376	Stage IIA	yes	no	0.568962	1
TCGA-55-7815	76	male	Alive	773	Stage IB	yes	yes	0.5510351	0
TCGA-97-A4M0	60	female	Alive	652	Stage IB	no	no	0.5699066	1
TCGA-44-5645	61	female	Alive	852	Stage IA	no	no	0.5555362	0
TCGA-75-7030	'--	male	Alive	'--	Stage IIB	yes	no	'--	'--
TCGA-62-A46S	73	male	Dead	1653	Stage IB	yes	no	0.592719	1
TCGA-86-A4JF	56	male	Dead	737	Stage IIB	yes	no	0.5668562	1
TCGA-05-5715	69	female	Alive	62	Stage IB	not reported	not reported	0.5816142	1
TCGA-95-7947	67	male	Alive	477	Stage IA	no	no	0.5585319	0
TCGA-50-6593	49	female	Dead	336	Stage IIIA	no	yes	0.5715712	1
TCGA-44-2666	43	male	Dead	97	Stage IB	no	no	0.5700393	1
TCGA-50-5051	42	female	Dead	478	Stage IIIA	yes	yes	0.5721469	1
TCGA-J2-A4AG	66	female	Alive	988	Stage IA	no	no	0.5996721	1
TCGA-55-8087	59	female	Alive	462	Stage IB	no	no	0.5607185	0
TCGA-97-A4LX	81	male	Alive	614	Stage IB	no	no	0.5656953	1
TCGA-55-A493	54	female	Alive	28	Stage IB	not reported	not reported	0.5543121	0
TCGA-62-A472	70	male	Alive	910	Stage IIB	no	yes	0.5607285	0
TCGA-80-5611	'--	male	Alive	2595	Stage IB	no	no	0.5331722	0
TCGA-55-6985	58	female	Alive	1233	Stage IB	no	no	0.5422948	0
TCGA-J2-A4AE	77	female	Alive	1079	Stage IA	no	no	0.5848765	1
TCGA-78-7542	56	male	Dead	321	Stage IB	not reported	not reported	0.5532491	0
TCGA-55-6543	60	female	Alive	435	Stage IA	no	no	0.554425	0
TCGA-97-7938	76	female	Dead	18	Stage IA	no	no	0.5693893	1
TCGA-05-4432	66	male	Alive	761	Stage IIB	yes	no	0.5648964	1
TCGA-93-7348	75	female	Alive	531	Stage IA	no	no	0.5810143	1
TCGA-78-7147	67	female	Dead	586	Stage IIB	no	no	0.5592689	0
TCGA-78-7148	71	male	Dead	626	Stage IIB	no	no	0.5578725	0
TCGA-35-3615	57	male	Alive	14	Stage IB	not reported	not reported	0.5662984	1
TCGA-91-A4BC	59	male	Alive	44	Stage IIA	not reported	not reported	0.5521274	0
TCGA-44-7671	64	male	Alive	889	Stage IB	no	no	0.5766672	1
TCGA-50-5932	75	male	Dead	1235	Stage IIB	yes	no	0.5687134	1
TCGA-38-6178	70	female	Alive	448	Stage IIIA	yes	no	0.5440286	0
TCGA-67-3770	70	female	Alive	610	Stage IA	not reported	not reported	0.5753605	1
TCGA-93-7347	76	female	Alive	683	Stage IA	no	no	0.5843658	1
TCGA-55-6984	71	female	Dead	760	Stage IIB	yes	no	0.5587495	0
TCGA-69-8254	85	male	Alive	409	'--	no	no	0.5803483	1
TCGA-55-6712	71	male	Dead	171	Stage IIA	yes	no	0.565249	1
TCGA-38-4630	75	female	Dead	1073	Stage IB	no	yes	0.5396715	0
TCGA-4B-A93V	52	female	Dead	300	Stage IA	no	yes	0.5495414	0
TCGA-86-8668	61	female	Alive	423	Stage IA	no	no	0.5677182	1
TCGA-MP-A4TI	72	male	Dead	429	Stage IIA	yes	yes	0.5549173	0
TCGA-44-2661	69	female	Alive	1159	Stage IA	no	no	0.5717285	1
TCGA-50-5941	55	female	Alive	1474	Stage IIIA	yes	no	0.5519153	0
TCGA-44-2657	74	female	Alive	1351	Stage IB	no	no	0.5659789	1
TCGA-97-8175	55	female	Alive	551	Stage IB	yes	yes	0.5795658	1
TCGA-69-7978	59	male	Alive	134	Stage IIB	no	no	0.5477447	0
TCGA-05-4405	74	female	Alive	610	Stage IB	no	no	0.5650669	1
TCGA-55-8505	62	male	Alive	440	Stage IIIA	yes	no	0.5634921	1
TCGA-49-4510	51	female	Dead	896	Stage IIB	yes	yes	0.577581	1
TCGA-64-1678	70	female	Alive	1189	'--	yes	no	0.5446711	0
TCGA-MP-A4TK	56	female	Dead	582	Stage IIB	yes	yes	0.5776392	1
TCGA-62-A46V	78	female	Alive	2199	Stage IB	no	no	0.5738275	1
TCGA-44-A4SS	73	male	Alive	415	Stage IA	no	no	0.5767445	1
TCGA-05-4250	79	female	Dead	121	Stage IIIA	not reported	not reported	0.5510857	0
TCGA-50-5044	72	female	Dead	624	Stage IIIB	yes	yes	0.541406	0
TCGA-64-1676	58	male	Alive	1728	Stage IA	no	no	0.5485778	0
TCGA-44-2665	55	female	Alive	1301	Stage IIB	yes	no	0.5367555	0
TCGA-78-7155	68	male	Dead	1171	Stage IB	not reported	not reported	0.5507086	0
TCGA-97-7937	65	male	Alive	564	Stage IB	no	no	0.5579236	0
TCGA-64-5774	60	male	Alive	2676	Stage IB	no	no	0.5465409	0
TCGA-78-7220	53	female	Dead	807	Stage IIIA	no	yes	0.5580628	0
TCGA-78-7167	77	male	Dead	2681	Stage IV	no	no	0.5813127	1
TCGA-55-8616	58	female	Alive	48	Stage IB	not reported	not reported	0.56174	0
TCGA-55-7570	60	male	Alive	824	Stage IA	no	no	0.552109	0
TCGA-78-7146	71	female	Dead	173	Stage IIIA	no	no	0.5407377	0
TCGA-44-3398	77	female	Alive	1163	Stage IA	no	no	0.5747421	1

Appendix Table S9. Clinical information of CPTAC-LUAD

case_submitter_id	gender	ajcc_pathologic_stage	age_at_diagnosis	vital_status	OSdays	Hsa00564	KM_Group
C3N-02672	female	Stage III	22056	Alive	1425	0.565957	1
C3N-02088	male	Stage IB	22039	Alive	430	0.560864	0
C3N-03205	female	Stage IIIA	15778	Alive	749	0.546226	0
C3L-03679	male	Stage IA	26038	Alive	1343	0.595395	1
C3L-02643	male	Stage IB	24797	Dead	1023	0.554756	0
C3L-03268	female	Stage IA	28668	Alive	11	0.573314	1
C3L-00412	male	Stage III	23597	Dead	162	0.558652	0
C3L-00893	male	Stage IB	26209	Alive	23	0.582498	1
C3N-02572	male	Stage IIA	18003	Alive	1135	0.557987	0
C3N-00560	male	Stage IIIA	20372	Alive	378	0.558739	0
C3N-02721	male	Stage IB	19893	Alive	324	0.562998	0
C3N-02146	female	Stage IIIA	25450	Not Reported	NA	'--	'--
C3L-04037	male	Stage IV	18998	Dead	251	0.550044	0
C3N-00737	male	Stage IA	26067	Alive	384	0.562728	0
C3L-00001	female	Stage IIA	22455	Alive	1837	0.546561	0
C3N-02149	male	Stage IIB	23517	Alive	413	0.59038	1
C3N-02929	male	Stage IA3	21205	Alive	1076	0.577519	1
C3L-02957	female	Stage IIB	20750	Alive	57	0.555846	0
C3N-01071	male	Stage IB	17831	Alive	44	0.559321	0
C3L-03717	female	Stage I	19827	Alive	167	0.563311	0
C3L-00913	male	Stage IIIA	24378	Dead	73	0.553195	0
C3L-00093	female	Stage IB	24301	Dead	1703	0.571111	1
C3N-02089	male	Stage IB	27910	Alive	403	0.575744	1
C3N-00579	male	Stage IB	24755	Alive	477	0.555351	0
C3N-02242	female	Stage IIIA	17416	Not Reported	NA	'--	'--
C3N-00549	male	Stage IB	21402	Alive	791	0.553348	0
C3L-00009	male	Stage IA	27968	Dead	577	0.559282	0
C3N-02145	female	Stage IB	24986	Alive	405	0.562824	0
C3N-02451	female	Stage IIB	28546	Alive	1735	0.577888	1
C3N-03212	female	Stage IIIA	25659	Alive	767	0.56599	1
C3L-02513	male	Stage IA	27012	Alive	1336	0.572502	1
C3L-00446	male	Stage IIA	26653	Dead	264	0.551658	0
C3N-03238	male	Stage IIA	17380	Alive	413	0.588059	1
C3L-02350	male	Stage IA	25374	Alive	1950	0.562807	0
C3N-01405	male	Stage IB	21609	Alive	418	0.556377	0
C3N-00217	female	Stage IB	21854	Alive	668	0.576549	1
C3L-00279	female	Stage I	26600	Alive	606	0.563818	0
C3L-02834	female	Stage IB	24663	Alive	205	0.567313	1
C3N-02290	male	Stage I	18896	Dead	414	0.579606	1
C3L-02165	male	Stage IIA	23666	Alive	6	0.58265	1
C3L-04040	male	Stage IIIA	28241	Alive	23	0.581291	1
C3L-02961	female	Stage I	25640	Alive	1424	0.586356	1
C3N-01823	male	Stage IIIA	23322	Dead	351	0.556648	0
C3N-02948	male	Stage IA	24802	Alive	1084	0.591012	1
C3N-02718	male	Stage IA	25231	Alive	4	0.555801	0
C3L-00263	male	Stage IIB	29164	Alive	1157	0.562987	0
C3L-02967	male	Stage I	27172	Alive	1519	0.592585	1
C3N-00578	male	Stage IIIA	17824	Dead	24	0.549687	0
C3N-02828	female	Not Reported	'--	Not Reported	NA	'--	'--
C3L-04759	female	Stage II	24773	Alive	6	0.566539	1
C3L-02508	male	Stage IIA	23855	Alive	1013	0.57381	1
C3L-00140	male	Stage IA	25869	Alive	1802	0.562756	0
C3N-02529	female	Stage IIB	29049	Dead	849	0.558377	0
C3N-04180	female	Stage IB	25948	Dead	709	0.562318	0
C3N-02424	male	Stage IIA	23634	Alive	355	0.551888	0
C3N-02282	male	Stage IIIA	23227	Alive	1831	0.536901	0
C3L-02958	male	Stage I	27124	Alive	1432	0.581543	1
C3N-01408	male	Stage IIIA	21281	Alive	406	0.583885	1
C3N-01417	male	Stage IIB	23444	Alive	434	0.575507	1
C3N-00550	female	Stage IIB	19107	Alive	401	0.556719	0
C3N-03765	male	Stage IA	22260	Dead	827	0.567685	1
C3L-03976	male	Stage IIIA	23885	Dead	301	0.561917	0
C3L-02365	female	Stage IIB	22934	Dead	627	0.580661	1
C3N-02922	male	Stage IIIA	20077	Not Reported	NA	'--	'--
C3N-01022	male	Stage IIIA	20195	Alive	396	0.577614	1
C3N-01074	male	Stage IB	20803	Dead	229	0.557309	0
C3L-01924	female	Stage IA	25001	Alive	1133	0.565417	1
C3N-00433	male	Stage IB	21129	Alive	60	0.559873	0
C3N-02230	female	Stage IB	24043	Not Reported	NA	'--	'--
C3L-02345	female	Stage IB	22572	Alive	1796	0.554995	0
C3L-01683	male	Stage IB	28432	Dead	1046	0.564667	0
C3N-03052	male	Stage IIB	23009	Alive	703	0.552472	0
C3N-00738	female	Stage IA	25891	Not Reported	NA	'--	'--
C3L-03642	male	Stage IC	23384	Alive	303	0.577772	1
C3L-01330	female	Stage IA	25076	Alive	1595	0.572133	1
C3L-01889	female	Stage IB	26205	Alive	1799	0.582634	1
C3N-02000	female	Stage IIA	16924	Alive	1086	0.559466	0
C3L-02515	female	Stage IIIA	25050	Alive	1818	0.573909	1
C3N-03074	female	Stage IIIA	19500	Alive	791	0.543221	0
C3L-00422	female	Stage IIA	12844	Alive	1916	0.57686	1
C3N-00180	male	Stage IIIA	20625	Dead	355	0.541937	0
C3N-01799	male	Stage IIA	29707	Alive	1545	0.569615	1
C3N-00547	male	Stage IIA	22202	Alive	474	0.57324	1
C3N-02715	female	Stage IIIA	13455	Alive	306	0.568382	1
C3L-04365	male	Stage I	23514	Alive	7	0.567409	1
C3N-02423	male	Stage IA	25686	Alive	695	0.565231	1
C3N-01488	male	Stage IB	19817	Alive	409	0.550442	0
C3L-03969	male	Stage II	28101	Dead	205	0.560655	0
C3N-01415	male	Stage IIIA	26008	Alive	792	0.578787	1
C3N-00546	male	Stage IIA	21464	Alive	382	0.554247	0
C3N-01021	female	Stage IIIA	16139	Alive	434	0.57035	1
C3N-03921	female	Stage IB	26682	Alive	3	0.555197	0
C3N-02193	female	Stage I	28999	Not Reported	NA	'--	'--
C3N-02764	male	Stage IIA	24321	Alive	1064	0.544095	0
C3L-00144	male	Stage IB	21487	Alive	578	0.557382	0
C3N-02142	male	Stage IB	23162	Alive	403	0.567137	1
C3N-02926	female	Stage IA3	18463	Alive	1077	0.565208	1
C3N-04382	female	Stage IB	17484	Alive	1130	0.537331	0

case_submitter_id	gender	ajcc_pathologic_stage	age_at_diagnosis	vital_status	OSdays	Hsa00564	KM_Group
C3L-02954	female	Stage IIIA	19171	Dead	637	0.54478	0
C3L-02549	male	Stage IIB	25447	Dead	447	0.57642	1
C3N-03202	male	Stage IA2	24932	Alive	756	0.553474	0
C3N-01409	female	Stage IB	22005	Alive	412	0.569327	1
C3L-00095	male	Stage IA	22863	Alive	1794	0.56851	1
C3L-01890	female	Stage IA	21246	Alive	671	0.578798	1
C3N-02588	male	Stage IIA	25225	Alive	1106	0.569697	1
C3N-02729	male	Stage IIA	16705	Dead	820	0.565337	1
C3N-01030	male	Stage IB	20772	Alive	443	0.561958	0
C3N-02950	female	Stage IB	26507	Dead	1359	0.578133	1
C3N-02586	male	Stage IIA	27040	Dead	30	0.570423	1
C3N-00556	male	Stage IIB	25462	Alive	394	0.545398	0
C3N-02067	female	Stage IIIA	13731	Alive	1081	0.57258	1
C3N-01016	male	Stage IIIA	20787	Alive	428	0.565576	1
C3N-01410	male	Stage IIB	23442	Alive	436	0.573216	1
11LU022	male	Stage IB	19370	Alive	7	0.552378	0
C3N-01023	male	Stage IIIA	21265	Alive	422	0.566951	1
C3L-04757	male	Stage IB	24886	Dead	1135	0.562272	0
C3L-02219	male	Stage IIIA	28347	Dead	931	0.575533	1
C3L-00510	female	Stage IA	29337	Alive	38	0.574585	1
C3N-00203	male	Stage IIB	26353	Not Reported	NA	'--	'--
C3L-02661	male	Stage IB	21632	Alive	8	0.547834	0
C3N-02923	male	Stage IIA	21896	Not Reported	NA	'--	'--
C3N-02141	female	Stage IIIA	26460	Alive	392	0.549039	0
C3N-04157	male	Stage IIIA	22497	Alive	432	0.562218	0
C3N-02234	male	Stage IIIA	24754	Not Reported	NA	'--	'--
C3L-02654	female	Stage IIIA	25783	Alive	34	0.544399	0
C3L-03462	male	Stage IIIA	22465	Alive	1436	0.551063	0
C3N-00552	female	Stage IB	18163	Alive	396	0.567142	1
C3N-01072	male	Stage IIIA	22957	Alive	449	0.543021	0
C3N-03911	female	Stage IB	25935	Alive	752	0.567964	1
C3N-03038	male	Not Reported	'--	Not Reported	NA	'--	'--
C3N-00545	male	Stage IA	21607	Alive	650	0.591968	1
C3N-01842	male	Stage IA	25527	Alive	681	0.537316	0
C3L-03721	male	Stage IA	20911	Alive	1341	0.577788	1
C3L-02559	female	Stage IB	23982	Alive	1764	0.577027	1
C3N-02143	male	Not Reported	'--	Not Reported	NA	'--	'--
C3L-03463	female	Stage IB	22953	Alive	1373	0.577406	1
C3N-01489	male	Stage IB	23810	Alive	434	0.568382	1
C3L-01862	male	Stage IIIA	29843	Dead	594	0.5501	0
11LU013	male	Stage IIIA	21627	Not Reported	NA	'--	'--
C3L-01632	female	Stage IIA	22145	Dead	717	0.565019	0
C3L-00444	female	Stage IA	22461	Alive	1919	0.583759	1
C3N-02090	male	Stage IB	22432	Alive	403	0.561944	0
C3N-03764	male	Stage IIB	25223	Dead	334	0.558022	0
C3N-02153	male	Stage IIIA	20241	Alive	402	0.560393	0
C3L-02348	female	Stage IIA	21302	Alive	1791	0.570462	1
C3N-03929	male	Stage IB	18638	Alive	33	0.566883	1
C3N-02158	female	Stage IIIA	19855	Alive	423	0.569748	1
C3N-02973	male	Stage IB	28611	Alive	1548	0.57335	1
C3N-02087	female	Stage IIIA	22433	Dead	303	0.556868	0
C3L-02129	male	Stage IV	20422	Alive	-11	0.566426	1
C3N-00559	female	Stage IA	20344	Alive	398	0.550759	0
C3L-00368	female	Stage IA	24636	Alive	141	0.583346	1
C3N-03057	male	Stage IIB	22417	Alive	634	0.559242	0
C3N-03210	male	Stage IA3	18827	Alive	664	0.580861	1
C3N-00167	male	Stage IIA	23543	Alive	842	0.584467	1
C3L-03271	female	Stage IB	25833	Alive	376	0.587602	1
C3N-02916	male	Stage IIIA	21891	Not Reported	NA	'--	'--
C3L-02616	male	Stage IIA	23275	Dead	244	0.533378	0
C3N-02758	female	Stage IIIA	28030	Alive	1079	0.577664	1
C3L-04033	male	Stage IIIA	14325	Alive	1405	0.546781	0
C3N-00959	male	Stage IB	24361	Alive	1758	0.594235	1
C3N-00580	male	Stage IB	20730	Alive	386	0.564336	0
C3N-03080	male	Stage IIA	24834	Alive	740	0.523929	0
C3N-03233	male	Stage IA	24895	Alive	295	0.544836	0
11LU035	male	Stage IA	21677	Alive	7	0.552862	0
C3N-01416	female	Stage IIIA	24540	Alive	434	0.568908	1
C3N-01413	female	Stage IB	22704	Alive	444	0.560511	0
C3N-02281	female	Stage IIA	23197	Dead	1436	0.572505	1
C3N-01419	male	Stage IB	21422	Alive	317	0.57587	1
C3N-01024	female	Stage IIB	24110	Alive	419	0.566873	1
C3N-02003	male	Stage IIIA	20728	Alive	353	0.572491	1
C3L-02601	male	Stage IIB	26133	Dead	201	0.584614	1
C3L-00973	male	Stage IB	27798	Alive	1435	0.559141	0
C3N-03073	male	Stage IIIA	25205	Alive	739	0.573277	1
C3N-00175	male	Stage IB	26470	Dead	308	0.545582	0
C3N-02928	male	Stage IIB	23733	Alive	1069	0.574222	1
C3N-02150	male	Stage IIB	18050	Alive	402	0.561366	0
C3N-00572	female	Stage IA	17745	Alive	431	0.574466	1
C3N-00169	male	Stage IB	21005	Alive	376	0.548909	0
C3N-02919	male	Stage IIIA	21151	Not Reported	NA	'--	'--
C3N-02587	female	Stage IA	21789	Alive	1111	0.578727	1
C3N-00294	male	Stage IIB	21155	Alive	1848	0.54797	0
C3L-00604	female	Stage IIIA	22335	Alive	1801	0.555653	0
C3N-02379	female	Stage IIB	20827	Dead	443	0.554054	0
C3N-03890	female	Stage IB	23096	Alive	1	0.554207	0
C3N-03225	male	Stage IIIA	20775	Alive	3	0.571385	1
C3N-04168	female	Stage IA	18727	Alive	417	0.553515	0
C3N-02433	female	Stage IA	25140	Alive	1137	0.583322	1
C3L-00080	male	Stage IB	21194	Alive	1805	0.552589	0
C3N-00574	male	Stage IB	14867	Alive	397	0.555216	0
C3N-00223	female	Stage I	25368	Alive	49	0.586248	1
C3N-04176	female	Stage IB	23173	Dead	289	0.562905	0
C3L-02169	male	Stage IIA	20732	Alive	1475	0.537886	0
C3L-02893	female	Stage I	26331	Alive	455	0.575124	1
C3N-02728	male	Stage IA	19982	Alive	13	0.55321	0
C3N-02582	male	Stage IIA	28227	Dead	888	0.564443	0
C3N-02422	female	Stage IV	20562	Dead	650	0.564358	0
C3L-03726	female	Stage IIA	28285	Alive	1365	0.584225	1

case_submitter_id	gender	ajcc_pathologic_stage	age_at_diagnosis	vital_status	OSdays	Hsa00564	KM_Group
C3L-01682	female	Stage IB	28669	Alive	1799	0.580405	1
C3L-03985	male	Stage IA	24731	Alive	1367	0.557454	0
C3N-02237	male	Stage II	25474	Not Reported	NA	'--	'--
C3N-00199	male	Stage IA	24010	Alive	1721	0.551194	0
C3N-02920	female	Stage IIIA	17859	Not Reported	NA	'--	'--
C3N-02155	male	Stage IIB	16568	Alive	423	0.535725	0
C3N-02380	male	Stage IIA	20483	Alive	1059	0.58357	1
C3N-00293	male	Stage IB	26774	Alive	1832	0.576462	1
C3L-00083	male	Stage IB	26127	Alive	1790	0.560307	0
C3L-03262	female	Stage IB	24929	Alive	97	0.592746	1
C3N-03054	male	Stage IA3	26640	Dead	317	0.558319	0
C3N-01019	male	Stage IB	20550	Alive	406	0.568559	1
C3N-04173	male	Stage IA	25564	Alive	1501	0.573167	1
C3L-00094	male	Stage IA	25560	Dead	889	0.565021	0
C3N-03063	male	Stage IB	22957	Dead	783	0.543989	0
C3L-02560	female	Stage IA	26748	Alive	1810	0.56361	0
C3N-00551	male	Stage IB	19986	Alive	391	0.57402	1
C3L-04031	female	Stage IIIA	26760	Alive	502	0.575866	1
C3N-02002	male	Stage IA	27900	Alive	355	0.571518	1
C3N-02757	male	Stage IA	25121	Alive	1053	0.559823	0
C3N-03420	female	Stage IIA	24141	Dead	1135	0.562962	0
C3N-02152	male	Stage IIB	22161	Not Reported	NA	'--	'--
C3N-00704	female	Stage IB	24887	Not Reported	NA	'--	'--
C3N-01414	male	Stage IB	21232	Alive	820	0.585951	1
C3N-03222	male	Stage IB	26630	Alive	263	0.565456	1
C3N-02144	male	Stage IIIA	26253	Not Reported	NA	'--	'--
C3N-02421	male	Stage IA	21515	Alive	1171	0.576299	1
C3N-02192	female	Stage I	24166	Not Reported	NA	'--	'--
C3N-02240	male	Stage IIB	18199	Not Reported	NA	'--	'--
11LU016	male	Stage IIIA	22733	Dead	445	0.557825	0
C3L-03984	male	Stage IIIA	21834	Alive	1086	0.562946	0

Appendix Table S10. The level of PE(18:0/18:1) and enrichment score of hsa00564 in cell assay.

	Human lung adenocarcinoma cells			Human normal lung epithelial cells		
	A549-1	A549-2	A549-3	BEAS-2B1	BEAS-2B2	BEAS-2B3
PE 18:0-18:1 level	4734.44	4050.92	5061.40	3591.54	3931.73	3301.97
Enrichenment Score of hsa00564	-0.066	-0.018	-0.052	0.096	0.123	0.023
Log2 PE 18:0-18:1 level	12.209	11.984	12.305	11.810	11.941	11.689

hsa00564: Glycerophospholipid metabolism
